# Evaluation of 16S rRNA genes sequences and genome-based analysis for identification of non-pathogenic *Yersinia*

**DOI:** 10.3389/fmicb.2024.1519733

**Published:** 2025-01-07

**Authors:** Angelina A. Kislichkina, Angelika A. Sizova, Yury P. Skryabin, Svetlana V. Dentovskaya, Andrey P. Anisimov

**Affiliations:** ^1^Department of Culture Collection, State Research Center for Applied Microbiology and Biotechnology, Obolensk, Russia; ^2^Department of Molecular Microbiology, State Research Center for Applied Microbiology and Biotechnology, Obolensk, Russia; ^3^Laboratory for Plague Microbiology, Especially Dangerous Infections Department, State Research Center for Applied Microbiology and Biotechnology, Obolensk, Russia

**Keywords:** *Yersinia*, genome, taxonomy, phylogeny, WGS, 16S rRNA, core SNPs, ANI

## Abstract

16S rRNA genes sequencing has been used for routine species identification and phylogenetic studies of bacteria. However, the high sequence similarity between some species and heterogeneity within copies at the intragenomic level could be a limiting factor of discriminatory ability. In this study, we aimed to compare 16S rRNA genes sequences and genome-based analysis (core SNPs and ANI) for identification of non-pathogenic *Yersinia*. We used complete and draft genomes of 373 *Yersinia* strains from the NCBI Genome database. The taxonomic affiliations of 34 genomes based on core SNPs and the ANI results did not match those specified in the GenBank database (NCBI). The intragenic homology of the 16S rRNA gene copies exceeded 99.5% in complete genomes, but above 50% of genomes have four or more variants of the 16S rRNA gene. Among 327 draft genomes of non-pathogenic *Yersinia*, 11% did not have a full-length 16S rRNA gene. Most of draft genomes has one copy of gene and it is not possible to define the intragenomic heterogenicity. The average homology of 16S rRNA gene was 98.76%, and the maximum variability was 2.85%. The low degree of genetic heterogenicity of the gene (0.36%) was determined in group *Y. pekkanenii*/*Y. proxima*/*Y. aldovae*/*Y. intermedia*/*Y. kristensenii*/*Y. rochesterensis*. The identical gene sequences were found in the genomes of the *Y. intermedia* and *Y. rochesterensis* strains identified using ANI and core SNPs analyses. The phylogenetic tree based on 16S rRNA genes differed from the tree based on core SNPs of the genomes and did not represent phylogenetic relationship between the *Yersinia* species. These findings will help to fill the data gaps in genome characteristics of deficiently studied non-pathogenic *Yersinia*.

## Introduction

The genus *Yersinia*, a member of the family *Yersiniaceae*, is currently composed of 26 species, including three human pathogens: the causative agent of plague, *Yersinia pestis*, and enteropathogenic *Yersinia enterocolitica* and *Yersinia pseudotuberculosis* (Mares et al., 2021; Le Guern et al., 2020). By reason of their medical significance, they have been well characterized, and data about their ecology, epidemiology, and molecular mechanisms of pathogenicity are available in many publications (Atkinson and Williams, 2016; Reuter et al., 2014). Other species of *Yersinia* are considered non-pathogenic for humans because they have not been shown to be associated with disease manifestation (Chen et al., 2010; Sulakvelidze, 2000). Nevertheless, the taxonomy of the genus *Yersinia* is evolving dynamically, and several novel species were recognized during WGS (Whole Genome Sequencing) investigations (Nguyen et al., 2020a; Nguyen et al., 2020b; Cunningham et al., 2019; Savin et al., 2019). Unlike the three human pathogens of *Yersinia*, other species have been less studied because most studies have focused on characterizing these *Homo sapiens* pathogens. As a result, our knowledge about non-pathogenic related species is very limited. Bacterial genome studies have shown that many pathogens can be separated from environmental, commensal, or zoonotic populations of microorganisms (Achtman et al., 1999; van Baarlen et al., 2007; Van Ert et al., 2007). The comprehensive studying of not clinically significant microorganisms is essential for understanding the evolution, ecology, virulence, and distribution of bacteria. The first step in studying of microorganism is properly to identify the species. Correct identification of clinical isolates is necessary for selecting optimal treatment strategies and determining the scope of public health measures.

16S rRNA sequencing has been used for decades for routine identification of bacterial isolates (Mignard and Flandrois, 2006). The advantage of the 16S rRNA gene over other genes is its presence in all known species of bacteria and archaea, as well as the existence of highly conserved regions, which made it possible to create universal primers suitable for ribotyping prokaryotes (Clarridge, 2004). This gene has become a widely used target for taxonomic and evolutionary studies of bacteria after the implementation of automatic genetic analyzers and the development of public databases containing a lot of nucleotide sequences of 16S rRNA genes (Cole et al., 2014; Caporaso et al., 2012). The 16S rRNA gene is approximately 1,500 bp long, and all known microorganisms have at least one copy of this gene. The nine hypervariable regions (V1–V9) and the conservative sequences separating them can be distinguished in the nucleotide sequence of the 16S rRNA gene (Abellan-Schneyder et al., 2021). Although 16S rRNA gene sequencing is widely used for microbe-species identification using WGS platforms, this method has several limitations and disadvantages (Johnson et al., 2019; Gonzalez et al., 2019; Muhamad Rizal et al., 2020). Results of identification could be unreliable in the case of using unsuitable primers, inadequate bioinformatic software, or outdated reference databases (Park and Won, 2018; Tatusova et al., 2015; Hsieh et al., 2022; Edgar, 2018). Among the factors limiting the discriminatory ability of this method are the high homology of the nucleotide sequences of this gene between several related genera and/or species and intragenic heterogenicity, i.e., polymorphisms between copies of 16S rRNA in the genome (Srinivasan et al., 2015; Rodriguez-R et al., 2018). In such cases, it is necessary to use additional genes or other methods to determine the species of bacteria.

The aim of our work was the evaluation of using 16S rRNA genes sequences and genome-based analysis for identification of non-pathogenic *Yersinia*. Complete genomes and WGS data from the NCBI Genome database were used in this study. We analyzed copies of the 16S rRNA gene in whole genomes to determine intragenic heterogenicity. The methods of the genome analysis such as core SNPs and ANI were used for species identification of *Yersinia*.

## Materials and methods

### Sample collection, DNA extraction, and whole-genome sequencing

A total of 33 non-pathogenic *Yersinia* strains were used in this study (Table 1). These strains are stored in the microorganism collection of the State Research Center for Applied Microbiology and Biotechnology (SRCAMB, Obolensk, Russia). Bacteria were originally collected as *Yersinia enterocolitica-*like, and were confirmed using microscopic examinations and biochemical identification tests. Before whole-genome sequencing, their species identifications were updated by matrix assisted laser desorption ionization (MALDI) Biotyper (Bruker, Germany).

**Table 1:** Data for whole genome-sequenced *Yersinia* strains.

Strain name	Sequencing technology	Assembly method^1^	Assembly accession no.	Total length, bp	Number of contigs	GC, %
*Y. aleksiciae* SCPM-O-B-8086 (J332)	Illumina MiSeq	Unicycler	JAASAK01	4290290	98	48.9
*Y. aleksiciae* SCPM-O-B-8087 (216)	Genolab M	Unicycler	JAQISD01	4376356	70	48.9
*Y. bercovieri* SCPM-O-B-7607	Illumina MiSeq	SPAdes	PEHN01	4318780	65	49.1
*Y. entomophaga* SCPM-O-B-7183	IonTorrent	SPAdes	MWTM01	4223348	93	48.5
*Y. frederiksenii* SCPM-O-B-3986	IonTorrent	SPAdes	MWTK01	4837254	77	47.5
*Y. frederiksenii* SCPM-O-B-7604	IonTorrent	SPAdes	MWTI01	4860925	49	47.5
*Y. frederiksenii* SCPM-O-B-8031	IonTorrent	SPAdes	MWTJ01	4941177	247	47.6
*Y. intermedia* SCPM-O-B-7605	IonTorrent	SPAdes	MWTO01	4994784	128	47.6
*Y. intermedia* SCPM-O-B-8114 (334)	Genolab M	Unicycler	JAQISA01	4921277	86	47.6
*Y. intermedia* SCPM-O-B-8304 (335)	Genolab M	Unicycler	JAQIRZ01	4999792	201	47.6
*Y. intermedia* SCPM-O-B-8305 (H357/85)	Genolab M	Unicycler	JAQIRY01	4911663	187	48.0
*Y. intermedia* SCPM-O-B-3971 (680)	Genolab M	Unicycler	JAQIRX01	5070029	237	47.6
*Y. intermedia* SCPM-O-B-10208 (116 INT)	Genolab M	Unicycler	JAPFIH01	4994370	206	47.6
*Y. intermedia* SCPM-O-B-10209 (333)	Genolab M	Unicycler	JBHFPP01	4780507	75	47.5
*Y. kristensenii* SCPM-O-B-8022 (C-141)	Genolab M	Unicycler	JAQISE01	4461680	27	47.5
*Y. kristensenii* SCPM-O-B-5043 (672)	Genolab M	Unicycler	JAQIRW01	4726318	59	47.2
*Y. kristensenii* SCPM-O-B-8070 (C-189)	Genolab M	Unicycler	JAQIRV01	4749883	69	47.7
*Y. kristensenii* SCPM-O-B-8021 (C-140)	Genolab M	Unicycler	JAQIRU01	4429905	102	47.6
*Y. kristensenii* SCPM-O-B-8023 (C-142)	Genolab M	Unicycler	JAQIRT01	4396862	177	47.8
*Y. massiliensis* SCPM-O-B-8024 (C-143)	Illumina MiSeq	SPAdes	PEHM01	4968403	69	47.4
*Y. massiliensis* SCPM-O-B-8025 (C-145)	Illumina MiSeq	Unicycler	JAASAN01	4807158	43	47.6
*Y. massiliensis* SCPM-O-B-8026 (C-146)	Genolab M	Unicycler	JAQISG01	4876124	63	47.6
*Y. mollaretii* SCPM-O-B-7596 (846/98)	Illumina MiSeq	Unicycler	JAASAJ01	4618139	30	49.0
*Y. mollaretii* SCPM-O-B-7598	Illumina MiSeq	SPAdes	PEHO01	4697334	61	49.1
*Y. mollaretii* SCPM-O-B-7609	Illumina MiSeq	SPAdes	PGLU01	4572689	46	49.0
*Y. mollaretii* SCPM-O-B-7610 (282/86)	Illumina MiSeq	Unicycler	JAASAI01	4626674	55	49.4
*Y. mollaretii* SCPM-O-B-10207 (176–36)	Genolab M	Unicycler	JAVKVJ01	4683382	73	49.1
*Y. mollaretii* SCPM-O-B-7597 (H87/82)	Genolab M	Unicycler	JAQISH01	4662038	61	49.1
*Y. mollaretii* SCPM-O-B-8306 (333)	Genolab M	Unicycler	JAQIRS01	4618025	31	49.0
*Y. rohdei* SCPM-O-B-7599	Genolab M	Unicycler	MWTN01	4405646	90	47.1
*Y. ruckeri* SCPM-O-B-8085 (H528)	IonTorrent	SPAdes	PEHK01	3828999	75	47.4
*Y. ruckeri* SCPM-O-B-8298 (H529-36/85)	Illumina MiSeq	SPAdes	JAQISB01	3798834	117	47.4
*Y. rochesterensis* SCPM-O-B-9106 (C-191)	Genolab M	Unicycler	JAQISF01	4349686	71	47.2

1 SPAdes – v. 3.9.0; Unicycler v. 0.4.7.

Bacteria were grown at 28°C on nutrient medium 1 (SRCAMB, Obolensk, Russia). DNA from each strain was extracted using the DNA minikit (BIOFACT Co., Ltd., Korea) following the manufacturer’s instructions. DNA quality was assessed using a Qubit 3 Fluorometer with the QubitTM dsDNA HS Assay Kit (Invitrogen, USA). Whole genome sequencing was performed in 2017, 2020 and 2022 using the Torrent PGM platform (Life Technologies, USA), Illumina MiSeq instrument (Illumina, USA), MGISeq-2000 (MGI Tech Co., China), and Genolab M (GeneMind Biosciences, China).

For sequencing on the Torrent PGM platform, the Ion 318 chip kit and 400-bp chemistry were used (Life Technologies, USA); on platform Illumina MiSeq - the Nextera DNA Library Preparation Kit and MiSeq Reagent Kits v3 (Illumina, USA); on the Genolab M - the library preparation kit SG GM (Raissol Bio, Sesana, Russia) and GenoLab M Sequencing Set V 1.0, FCM 300 cycles (GeneMind Biosciences, China); on MGISeq-2000 - MGIEasy FS DNA Library Prep Kit and MGI-Seq 2000RS High-throughput sequencing kit PE200 (MGI Tech Co., China).

The raw reads were *de novo* assembled using assemblers SPAdes v. 3.9.0 and Unicycler v. 0.4.7 with default settings, which included primary filtering and quality control (Bankevich et al., 2012; Wick et al., 2017). The draft genomes were deposited in GenBank database. Annotation was carried out by NCBI Prokaryotic Genome Annotation Pipeline (PGAP) v. 5.3. Information on the assembly accession number in NCBI Genome database, total length, number of contigs and GC percentage is shown in Table 1.

### Bacterial genomes

The genomes of 33 non-pathogenic *Yersinia* strains performed in this study and all complete and draft genomes of non-pathogenic *Yersinia* downloaded at NCBI Genome (September, 2022) were included in the investigation. Finally, the genomes of 368 strains of non-pathogenic *Yersinia* (*Y. aldovae* – 11, *Y. aleksiciae* – 11, *Y. alsatica* – 10, *Y. artesiana* – 4, *Y. bercovieri* – 17, *Y. canariae* – 3, *Y. entomophaga* – 2, *Y frederiksenii* – 37, *Y. hibernica* – 2, *Y. intermedia* – 41, *Y. kristensenii* – 38, *Y. massiliensis* – 15, *Y. mollaretii* – 27, *Y. nurmii* – 1, *Y. pekkanenii* – 2, *Y. proxima* – 10; *Y. rochesterensis* – 8; *Y. rohdei* – 12; *Y. ruckeri* – 99; *Y. similis* – 9, *Y. thracica* – 4, *Y. vastinensis* – 5), as well as genomes of *Y. enterocolitica* 8081, *Y. enterocolitica* subsp*. palearctica* Y11, *Y. pseudotuberculosis* IP 32953, *Y. pestis* CO92, and *Y. wautersii* WP-931201 were studied. The data are available in the NCBI Genome database, and accession numbers are provided in Supplementary Table S1.

### Phylogenetic analysis of *Yersinia* genomes based on core SNPs

The core SNPs were determined using the Snippy 4.6.0 software with default settings.^1^ Visualization of the phylogenetic trees were performed using the Neighbor joining algorithm FigTree v. 1.4.4^2^ and SplitsTree4^3^ using NJ method.

### Determination of ANI

Average nucleotide identity (ANI) values were determined using the FastANI software with default settings (Jain et al., 2018). Statistical calculations were performed using MS Office Excel.

### Analysis of 16S rRNA gene in *Yersinia* genomes

The 16S rRNA gene searches were performed using BLAST. For phylogenetic analysis only full-length genes were selected from the genomes. The alignment was performed using MEGA11 with the ClustalW algorithm (Tamura et al., 2021) with default settings. The phylogenetic tree was constructed using the Neighbor joining algorithm in MEGA11 software.

## Results

### Diversity of copies 16S rRNA gene in whole genomes

The copies of 16S rRNA genes in complete genomes from the GenBank database (NCBI) were compared to evaluate the intragenomic heterogenicity of the 16S rRNA genes of *Yersinia*. Note that the complete genomes of *Y. wautersii*, *Y. vastinensis*, *Y. artesiana*, *Y. proxima*, *Y. pekkanenii*, *Y. thracica*, and *Y. nurmii* are not available from the NCBI database. Only one or a few complete genomes are available for other *Yersinia* species. Among the non-pathogenic *Yersinia* species for humans and other warm-blooded animals, *Y. ruckeri* is most represented among sequenced genomes because it is a cause of a serious septicemic bacterial disease in salmonid fish.

The complete genomes of *Yersinia*, their accession numbers, platforms on which WGS was performed, and assembly software are provided in Supplementary Table S2. The genomes of *Yersinia* species contain seven copies of the 16S rRNA gene, except for *Y. pestis*, which contains six copies per genome. The analysis of 16S rRNA gene copies in the complete genomes of *Yersinia* is shown in Table 2.

**Table 2:** Analysis of 16S rRNA gene copies in complete genomes of *Yersinia*.

Strain name	Number of gene variants	Number of variable nucleotides	Number of in/del	Number of in/del and SNP between the most different copies	Homology between the most different copies
*Y. aldovae* 670–83	6	13	2	14	99.09
*Y. frederiksenii* FDAARGOS_417	5	6	2	6	99.61
*Y. frederiksenii* FDAARGOS_418	5	3	1	4	99.74
*Y. frederiksenii* Y225	7	29	2	20	98.70
*Y. bercovieri* ATCC 43970	4	7	–	6	99.61
*Y. rohdei* YRA	3	0	4	4	99.74
*Y. intermedia* FDAARGOS_730	5	8	1	6	99.61
*Y. intermedia* FDAARGOS_729	4	8	–	6	99.61
*Y. intermedia* N6/293	5	7	–	7	99.55
*Y. intermedia* Y228	3	6	–	5	99.68
*Y. intermedia* NCTC11469	5	7	–	7	99.55
*Y. intermedia* FDAARGOS_358	2	1	–	1	99.94
*Y. mollaretii* ATCC 43969	4	3	–	1	99.94
*Y. rochesterensis* ATCC 33639	4	4	2	3	99.81
*Y. kristensenii* 2012N–4030	3	2	–	2	99.87
*Y. rochesterensis* Y231	3	2	2	3	99.81
*Y. massiliensis* 2011N–4075	3	2	–	1	99.94
*Y. massiliensis* GTA	3	2	–	1	99.94
*Y. entomophaga* MH96	5	9	1	6	99.61
*Y. alsatica* SCPM–O–B–7604	1	0	–	0	100
*Y. similis* 228	2	1	–	1	99.94
*Y. rochesterensis* ATCC BAA–2637	2	1	–	1	99.94
*Y. aleksiciae* 159	2	1	–	1	99.94
*Y. hibernica* CFS1934	1	0	–	0	100
*Y. canariae* NCTC 14382	6	5	4	6	99.61
*Y. pestis* CO92	5	4	–	1	99.94
*Y. pseudotuberculosis* IP 32953	5	8	–	5	99.68
*Y. enterocolitica* 8,081	3	3	–	3	99.81
*Y. enterocolitica* subsp*. palearctica* Y11	5	9	–	10	99.35
*Y. ruckeri* KMM821	1	0	–	0	100
*Y. ruckeri* QMA0440	1	0	–	0	100
*Y. ruckeri* 17Y0412	4	2	2	3	99.81
*Y. ruckeri* 17Y0189	6	2	4	2	99.87
*Y. ruckeri* SC09	2	1	–	1	99.94
*Y. ruckeri* 17Y0153	4	2	1	1	99.94
*Y. ruckeri* 17Y0155	4	2	2	2	99.87
*Y. ruckeri* 17Y0159	7	3	8	4	99.74
*Y. ruckeri* 17Y0414	2	1	–	1	99.94
*Y. ruckeri* 17Y0163	2	1	–	1	99.94
*Y. ruckeri* NHV_3758	2	1	–	1	99.94
*Y. ruckeri* 17Y0157	4	0	3	1	99.94
*Y. ruckeri* Big Creek 74	4	1	3	2	99.87
*Y. ruckeri* 16Y0180	5	3	2	3	99.81
*Y. ruckeri* 17Y0161	3	2	–	1	99.94
*Y. ruckeri* YRB	6	2	8	3	99.81

Among investigated *Yersinia* were observed different number of 16S rRNA gene copy variants in the complete genomes. In two genomes (*Y. frederiksenii* Y225 and *Y. ruckeri* 17Y0159) of each copy of gene was unique. Six, five and four variants of 16S rRNA gene had 4, 10 and 9 genomes, respectively. Two and three variants of 16S rRNA gene had 8 genomes each. Interestingly, only four genomes (*Y. alsatica* SCPM-O-B-7604, *Y. hibernica* CFS1934, *Y. ruckeri* KMM821, *Y. ruckeri* QMA0440) among the complete sequences contained only one 16S rRNA gene variant. The nucleotide differences are more common than the formation of insertions or deletions. Ins/del were detected in 19 of 45 complete genomes of *Yersinia*. Number of in/del and SNP between the most different copies were varied, but in 25 genomes it was ≤3. More than 10 mismatches between the most different copies were observed in genome *Y. frederiksenii* Y225 (20) and in *Y. aldovae* 670–83 (14). In the rest genomes, number of in/del and SNP was ≥4 and ≤ 10 between the most different copies.

**Figure 1:**
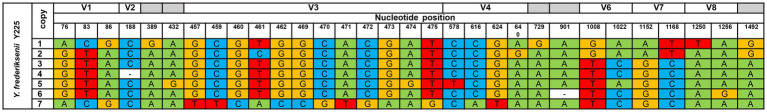
Comparison of the copies 16S rRNA gene *Y. frederiksenii* Y225. Coordinates of copies in chromosome (CP009364): 1 – 1663173–64703 bp; 2 – 3873144–3874674 bp; 3 – 3178807–3180337 bp; 4 – 2408072–2409601 bp; 5 – 3499799–3501329 bp; 6 – 3139875–3141404 bp; 7 – 3333051–3334581 bp. V1, V2, V3, V4, V6, V7, V8 – variable regions. Nucleotide positions were determined according to the *E. coli* gene nomenclature (Church et al., 2020).

Pairwise comparison of gene copy sequences in one genome defined homology exceeding 99% for all strains, except for *Y. frederiksenii* Y225. The mismatch of 16S rRNA copies of this genome is presented in Figure 1. The total nucleotide mismatches of 31 points were detected, 29 SNPs and two insertion/deletion (ins/del). 26 points are in variable regions that are commonly used for species and genus identification; more than half of the mismatches (20) were in regions V1-V4, which are the most variable, and sequencing of these gene regions is most often used for routine identification of microorganisms. Each 16S rRNA copy in the *Y. frederiksenii* Y225 genome is unique. The maximum number (20 SNPs and ins/del) of differences between copies of the gene was also defined in this genome.

### Cluster analysis based on 16S rRNA gene sequence

For the analysis, only full-length genes were selected. From the complete and draft genomes, up to 10 scaffolds were taken of all copies of the gene. In most of the draft genomes one copy of the 16S rRNA gene was present; two full-length copies were identified in only four draft genomes. The full-length assembled 16S rRNA gene was absent in 36 drafts (9%). The total number of 16S rRNA genes (644 sequences) was aligned using MEGA11 (ClustalW algorithm). Each species of the genus *Yersinia* was represented by at least one copy of the 16S rRNA gene. Pairwise comparisons of the 16S rRNA genes are provided in Supplementary Table S3. A phylogenetic tree was constructed using the Neighbor joining algorithm. The expanded phylogenetic tree is presented in Supplementary Figure S1. The branches composed of identical genes or genes with some nucleotide differences were compressed (Supplementary Figure S2). As a result, the sequences of 16S rRNA genes formed six large clades, namely – 1a, 1b, 2a, 2b, 3a, and 3b, on the phylogenetic tree.

Clade 1a comprises two branches, the first includes all sequences of *Y. ruckeri* 16S rRNA and, the second contains part of the *Y. kristensenii* 16S rRNA gene sequences. The average homology rates inside the branches is 99.94 and 99.91%, respectively. Group 1b comprises of three branches. The first branch included all 16S rRNA sequences of *Y. bercovieri* and *Y. aleksiciae*, with an average homology of 99.66%. The second branch comprises all 16S rRNA genes of *Y. mollaretii*, with an average homology of 99.93%. The third branch includes the 16S rRNA sequences of several species: *Y. pekkanenii*, *Y. proxima*, *Y. aldovae*, *Y. intermedia*, *Y. kristensenii*, and *Y. rochesterensis*, with an average 16S rRNA homology of 99.64%. A few strains of *Y. intermedia* and *Y. rochesterensis* have the identical gene sequences.

Clade 2a included all 16S rRNA sequences of *Y. massiliensis*, and the average homology was 99.85%. The clade 2b are formed by three branches. The first branch included 16S rRNA sequences of *Y. similis*, *Y. pseudotuberculosis*, *Y. pestis*, and *Y. wautersii*, with an average homology of 99.76%. The second branch contains 16S rRNA sequences of *Y. alsatica*, with an average homology of 99.71%. The third branch includes *Y. frederiksenii* 16S rRNA sequences; the average gene homology was 99.91%.

Clade 3a contains two branches (*Y. entomophaga/Y. nurmii* and *Y. vastinensis/Y. frederiksenii*) and a separate 16S rRNA sequence from the complete genome of *Y. frederiksenii* Y225. Average homology of the group – 99.5%.

Clade 3b includes the 16S rRNA of *Y. rohdei/Y. thracica*, *Y. canariae*, *Y. artesiana*, *Y. enterocolitica*, and *Y. hibernica*/*Y. kristensenii*, with separate located sequences of *Y. rohdei* 68/02 and *Y. rohdei* 3,343, as well as the 16S rRNA gene of *Y. aldovae* IP07632. The average 16S rRNA homology in clade 3b was 99.05%.

Analysis of the 16S rRNA genes revealed identical sequences in some genomes of strains related to different species, according to NCBI data. The results are listed in Table 3.

**Table 3:** Sequences of identical 16S rRNA genes in the genomes of different *Yersinia* species.

Clade	Strains
1b	*Y. intermedia*: IP39994, IZSPB_Y97, 93/02, FDAARGOS_729, FDAARGOS_730, NCTC11469, FDAARGOS_358, SCPM-O-B-10209, N6/293 *Y. kristensenii*: IP28581, MGYG-HGUT-02462, OK6311, FE80982 *Y. rochesterensis*: IP37484, IP35638, IP38810, SCPM-O-B-9106 (C-191), Y231, ATCC BAA-2637 *Y. frederiksenii*: Y225
	*Y. intermedia*: FCF130, FCF84 *Y. rochesterensis*: IP38921, ATCC BAA-2637, Y231 ATCC 33639
	*Y. proxima*: IP39432, IP39924, IP38191 *Y. intermedia*: 58735
2a	*Y. massiliensis*: 2011 N-4075, GTA, SCPM-O-B-8024, SCPM-O-B-8026 (C-146), IZSPB_Y116, SCPM-O-B-8025 (C-145), IP39269, IP34847, IP34847 *Y. frederiksenii*: FDAARGOS_417, FE80988
	*Y. massiliensis*: IZSPB_Y100, CCUG 53443, CIP109351 *Y. intermedia*: R148
3a	*Y. vastinensis*: IP38006, IP38831 *Y. frederiksenii*: CFSAN060535
2b	*Y. pseudotuberculosis* IP 32953 *Y. pestis* CO92
	*Y. alsatica* SCPM-O-B-7604 *Y. frederiksenii* SCPM-O-B-7604

### Identification and phylogenetic analysis of *Yersinia* genomes based on core SNPs

**Figure 2:**
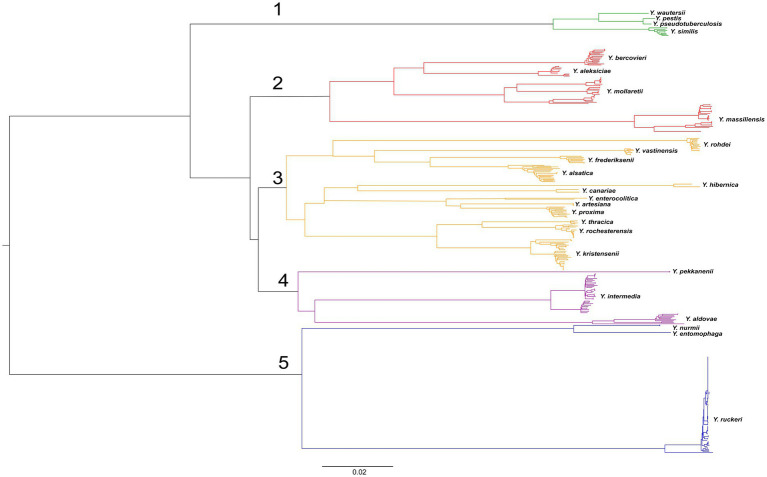
Phylogenetic tree of *Yersinia* genomes. A phylogenetic tree was conducted using the neighbor-joining (NJ) algorithm based on 9,494 core SNPs generated by the Snippy 4.6.0 software. This tree was rooted using midpoint option. The groups of genomes observed in the tree were identified according to the species names of the type strains. The scale bar shows the expected number of substitutions per site. Bar, 0.02 substitutions per nucleotide position.

The determination of core SNPs in 368 genomes of non-pathogenic *Yersinia* and genomes of *Y. enterocolitica* subsp*. enterocolitica* 8081, *Y. enterocolitica* subsp*. palearctica* Y11, *Y. pseudotuberculosis* IP 32953, *Y. pestis* CO92, and *Y. wautersii* WP-931201 was performed using Snippy 4.6.0 software (Snippy, 2015). A phylogenetic tree based on the 9,494 core SNPs was built using neighbor joining algorithm in the FigTree 1.4.3 software (FigTree 1.4.3, 2017) (Figure 2). The detailed phylogenetic tree is presented in Supplementary Figure S3.

The genomes formed five clades on the tree. The first clade includes the genomes of the closely related species *Y. pseudotuberculosis*, *Y. pestis*, *Y. similis*, and *Y. wautersii*. This clade can be reliably distinguished from other *Yersinia* species. The second clade consists of the species *Y. aleksiciae*, *Y. bercovieri*, *Y. mollaretii*, and the neighboring species *Y. massiliensis* on long, distant branches. The third clade can be distinguished on two branches.

The first included species *Y. frederiksenii*, *Y. vastinensis*, *Y. alsatica*, and *Y. rohdei*. The second branch comprises two groups: *Y. enterocolitica* together with its related species *Y. proxima*, *Y. artesiana*, and *Y. canariae*, and the closely related species *Y. kristensenii*, *Y. thracica*, and *Y. rochesterensis*. The fourth clade comprises the separate branches of *Y. pekkanenii*, *Y. aldovae*, and *Y. intermedia*. The fifth clade significantly outlies the other clades and consists of two branches: the first is represented by genomes of *Y. ruckeri*, and the second composes of genomes of related species *Y. nurmii* and *Y. entomophaga*.

The species relationship of most genomes of the genus *Yersinia* was matched with those specified in the GenBank database (NCBI), but taxonomic inconsistencies in 34 bacterial genomes were determined (Table 4). The genomes of 25 *Yersinia* strains were deposited as *Y. frederiksenii*, but according to the results of the analysis of core SNPs, 12 were assigned as *Y. alsatica*, nine as *Y. massiliensis*, four as *Y. vastinensis*, and one as *Y. rochesterensis*. Six of the *Y. kristensenii* genomes were identified as *Y. rochesterensis* and one as *Y. hibernica*. One *Y. intermedia* genome was located between *Y. proxima* genomes in the phylogenetic tree, and another was located between *Y. massiliensis* genomes.

**Table 4:** Identification of mismatched *Yersinia* genomes.

	Strain name	GenBank accession no.	Species indicated in GenBank	The closest species by core SNPs and ANI	Average ANI comparing to genomes of species	
					Indicated in GenBank (%)	The closest by core SNPs (%)
1	FDAARGOS_417	CP023962	*Y. frederiksenii*	*Y. massiliensis*	83.45	98.05
2	FE80988	CQEN01	*Y. frederiksenii*	*Y. massiliensis*	83.46	98.03
3	FCF467	CGCI01	*Y. frederiksenii*	*Y. massiliensis*	83.44	98.03
4	IP23698	CPZP01	*Y. frederiksenii*	*Y. massiliensis*	83.39	98.15
5	112/02	CTKH01	*Y. frederiksenii*	*Y. massiliensis*	83.43	98.07
6	3400/83	CGCB01	*Y. frederiksenii*	*Y. massiliensis*	83.42	98.07
7	MGYG-HGUT-02465	CABMMF01	*Y. frederiksenii*	*Y. massiliensis*	83.42	98.07
8	120/02	CTJA01	*Y. frederiksenii*	*Y. massiliensis*	83.42	98.07
9	SCPM-O-B-3986	MWTK01	*Y. frederiksenii*	*Y. massiliensis*	83.45	98.14
10	FE80258	CPWN01	*Y. frederiksenii*	*Y. alsatica*	92.16	98.63
11	MGYG-HGUT-02467	CABMMI01	*Y. frederiksenii*	*Y. alsatica*	92.16	98.63
12	CFSAN060534	NHOK01	*Y. frederiksenii*	*Y. alsatica*	92.05	97.83
13	28/85	CQEP01	*Y. frederiksenii*	*Y. alsatica*	92.18	98.71
14	498/85	CQEC01	*Y. frederiksenii*	*Y. alsatica*	92.21	98.57
15	IP25924	CQBF01	*Y. frederiksenii*	*Y. alsatica*	92.13	98.57
16	22714/85	CQBO01	*Y. frederiksenii*	*Y. alsatica*	92.15	98.57
17	FCF343	CQBR01	*Y. frederiksenii*	*Y. alsatica*	92.14	98.61
18	RS-42	CQDT01	*Y. frederiksenii*	*Y. alsatica*	92.09	98.03
19	IP23047	CGBN01	*Y. frederiksenii*	*Y. alsatica*	92.13	98.60
20	SCPM-O-B-7604	MWTI01	*Y. frederiksenii*	*Y. alsatica*	92.17	98.71
21	SCPM-O-B-8031	MWTJ01	*Y. frederiksenii*	*Y. alsatica*	92.26	98.61
22	FCF00224	CQBW01	*Y. frederiksenii*	*Y. vastinensis*	87.02	99.56
23	3430	CQDU01	*Y. frederiksenii*	*Y. vastinensis*	87.01	99.54
24	FCF208	CQBX01	*Y. frederiksenii*	*Y. vastinensis*	87.02	99.63
25	CFSAN060535	NHOJ01	*Y. frederiksenii*	*Y. vastinensis*	87.02	99.61
26	Y225	CP009364	*Y. frederiksenii*	*Y. rochesterensis*	85.29	99.47
27	ATCC 33639	CP008955	*Y. kristensenii*	*Y. rochesterensis*	92.99	99.47
28	FE80982	CQAQ01	*Y. kristensenii*	*Y. rochesterensis*	93.02	99.43
29	MGYG-HGUT-02462	CABMMB01	*Y. kristensenii*	*Y. rochesterensis*	93.02	99.43
30	OK6311	CWJK01	*Y. kristensenii*	*Y. rochesterensis*	93.05	99.46
31	IP28581	CABHXY01	*Y. kristensenii*	*Y. rochesterensis*	93.14	99.34
32	CFSAN060539	NHOF01	*Y. kristensenii*	*Y. hibernica*	86.66	98.58
33	58735	CQER01	*Y. intermedia*	*Y. proxima*	84.58	99.18
34	R148	CWJI01	*Y. intermedia*	*Y. massiliensis*	83.37	96.62

### Identification of *Yersinia* genomes based on ANI

ANI was calculated pairwise for each of the 373 genomes (Supplementary Table S4).

The genomes were grouped according to an ANI value of 95–96% which is the bacterial species threshold (Riesco and Trujillo, 2024). The species identified in of the same 34 bacterial genomes did not match those specified in the GenBank database (NCBI). The taxonomic affiliations of these genomes based on core SNPs were determined according to the ANI results. The mean ANI value for each of the 34 genomes relative to the genomes of the species indicated in the NCBI database and the genomes of the species identified by core SNPs are shown in the Table 4.

**Figure 3:**
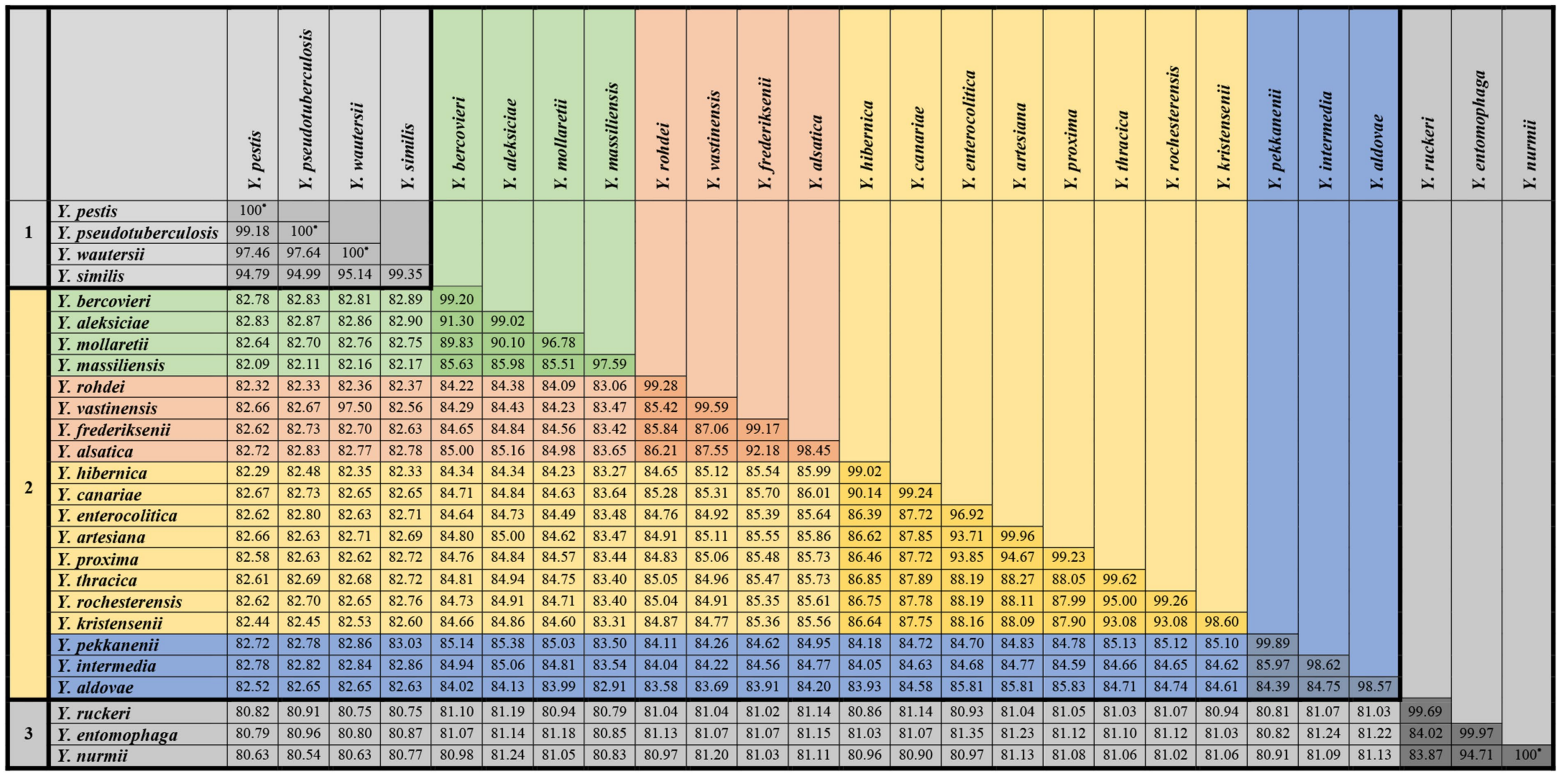
Average ANI values for all genomes of each species *Yersinia* were determined in relation to those strains belonging to other species.

The average ANI value for all genomes of each species was determined in relation to the genomes of strains belonging to other species (Figure 3). Generally, *Yersinia* can be divided into three groups according to ANI results. The first group consisted of *Y. ruckeri*, *Y. nurmii*, and *Y. entomophaga*, with the lowest ANI (80.27–81.90%) in relation to other *Yersinia* species. The ANI values between *Y. entomophaga* and *Y. nurmii* is very close to the species threshold (94.71%).

The second group, consisting of four species, *Y. pseudotuberculosis*, *Y. pestis*, *Y. wautersii*, and *Y. similis,* was distinguished from other *Yersinia* species. The ANI value between the genomes of *Y. pseudotuberculosis* IP 32953, *Y. pestis* CO92, and *Y. wautersii* WP-931201 is more 97%, which is above the species threshold. These species are sometimes integrated into the *Y. pseudotuberculosis* complex, and a high ANI value is a significant reason for combining these species into one complex. The ANI of *Y. similis* ranges from 94.71 to 95.20% relatively to *Y. pseudotuberculosis* complex, demonstrating a close relationship. The ANI values between this group and other *Yersinia* species fluctuated from 81.94 to 83.26%.

The third group included the remaining species with ANI values of 81.93–95.16% between them. In this group, three pairs of species had high ANI values, indicating closer genetic relationships. These pairs of species were *Y. aleksiciae* and *Y. bercovieri*; *Y. alsatica* and *Y. frederiksenii*; *Y. canariae* and *Y. hibernica*. The two trios of species *Y. enterocolitica*, *Y. artesiana*, and *Y. proxima*; *Y. kristensenii*, *Y. rochesterensis*, and *Y. thracica* have higher 90% ANI values, indicating a close relationship.

**Figure 4:**
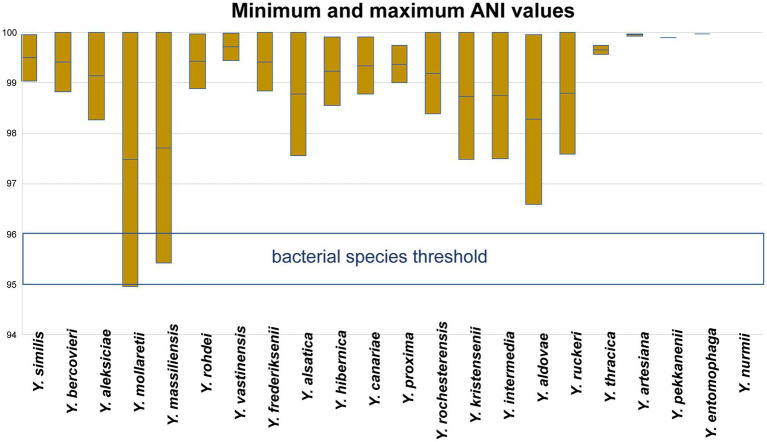
Minimum and maximum ANI values within *Yersinia* non-pathogenic species.

The minimum and maximum ANI values within each *Yersinia* species were evaluated (Figure 4). The minimum ANI values relevant to the bacterial species threshold were found between the genomes of *Y. mollaretii* and *Y. massiliensis* at 94.95 and 95.42%, respectively.

**Figure 5:**
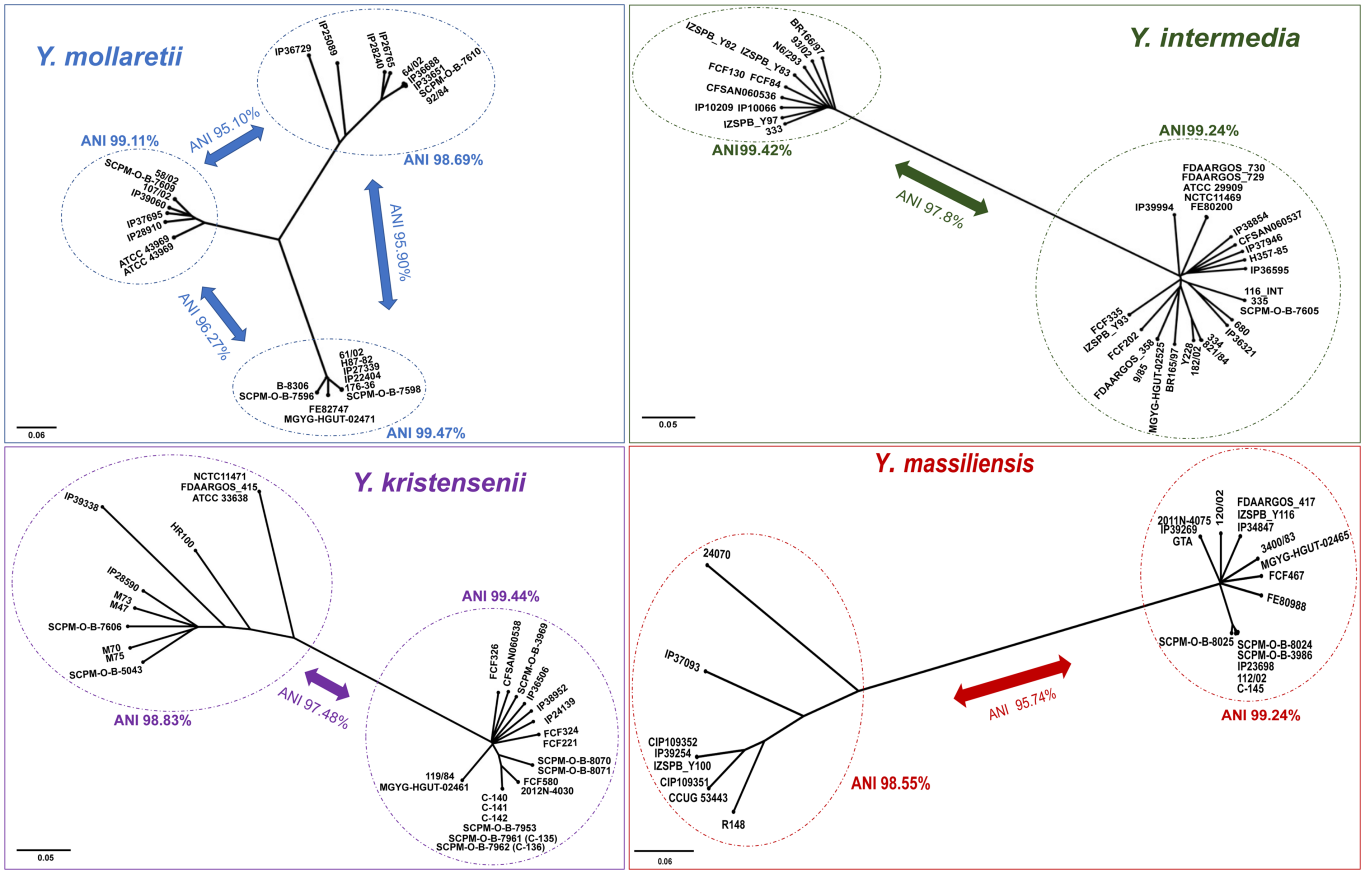
Phylogenetic trees of *Y. mollaretii*, *Y. massiliensis*, *Y. intermedia*, and *Y. kristensenii* showing the existence of the lineages into the species. A phylogenetic tree was conducted using the neighbor-joining (NJ) algorithm based on the core SNPs generated by the Snippy 4.6.0 software (*Y. mollaretii* – 259,428 SNPs, *Y. massiliensis* – 199,117 SNPs, *Y. intermedia* – 111,428 SNPs, and *Y. kristensenii* – 148,020 SNPs). The scale bar shows the expected number of substitutions per site. Bar, 0.05 or 0.06 substitutions per nucleotide position. The ANI values of the lineages are indicated.

A study of non-pathogenic *Yersinia* genomes using the core SNPs showed the existence of separate lineages (probable subspecies) within the species *Y. mollaretii*, *Y. massiliensis*, *Y. intermedia*, and *Y. kristensenii*. The separate phylogenetic trees (SplitsTree4 software, NJ method) (SplitsTree4, 1996) were constructed for these species, and ANI values were determined (Figure 5).

The comparison of the core SNPs showed that the genomes of *Y. mollaretii* and *Y. massiliensis* are divided into three and two lineages, respectively. The ANI values between the three *Y. mollaretii* lineages ranged from 95.10 to 96.27%, whereas the values within each lineage were higher (98.68%). The ANI value between two lineages of the *Y. massiliensis* genome was 95.74%, whereas the values within each lineage were 99.24 and 98.55%.

The genomes of species *Y. kristensenii* and *Y. intermedia* were divided into two lineages by the results of core SNPs. The ANI value between lineages of *Y. kristensenii* was 97.40%. The mean ANI value within the genomes of the first lineage was 99.44%, the second lineage is more heterogeneous, the mean ANI value was 98.83%. The genomes of *Y. intermedia* can also be divided into two lineages with mean ANI value of 97.80%. The ANI values within the first and second lineages are 99.24 and 99.42%, respectively.

### Comparison of identification based on the 16S rRNA gene, core SNPs, and ANI

According to the clustering results, some groups included strains belonging to different species, as indicated in the NCBI database. Thus, group 2a included all 16S rRNA sequences of *Y. massiliensis* strains, as well as 11 genes of *Y. frederiksenii* (7 gene copies from the complete genome of strain FDAARGOS_417, as well as one copy each from the draft genomes of strains - FE80988, FCF467, 120/02, SCPM-O-B-3986) and one gene from the draft genome of *Y. intermedia* R148. According to the core SNPs and ANI analyses, these strains belong to the *Y. massiliensis*.

Some genes of *Y. frederiksenii* strains CFSAN060534, 28/85, 22,714/85, IP23047, SCPM-O-B-7604, and SCPM-O-B-8031 were grouped with the genes of *Y. alsatica*. Genes of *Y. frederiksenii* strains 3,430, FCF208, CFSAN060535, and FCF224 were clustered with *Y. vastinensis* as well. Based on core SNPs and ANI analyses, these *Y. frederiksenii* strains belong to confirmed species. In the same way the strains *Y. kristensenii* CFSAN060539 (*Y. hibernica*), *Y. intermedia* R148 (*Y. massiliensis*) *Y. intermedia* 58735 (*Y. proxima*) were misidentified.

However, identical 16S rRNA gene sequences were identified for *Y. intermedia* and *Y. rochesterensis* in the two groups of strain. The first group included *Y. intermedia* (IP39994, IZSPB_Y97, 93/02, FDAARGOS_729, FDAARGOS_730, NCTC11469, FDAARGOS_358, SCPM-O-B-10209, N6/293), *Y. rochesterensis* (IP37484, IP35638, IP38810, SCPM-O-B-9106 (C-191), Y231, ATCC BAA-2637), and strains *Y. frederiksenii* Y225, *Y. kristensenii* (IP28581, MGYG-HGUT-02462, OK6311, FE80982). However, the strains *Y. frederiksenii* and *Y. kristensenii* belong to *Y. rochesterensis*, according to genome analysis. The second group of identical 16S rRNA gene sequences consisted of *Y. intermedia* FCF130, *Y. intermedia* FCF84, *Y. rochesterensis* IP38921, *Y. rochesterensis* ATCC BAA-2637, *Y. rochesterensis* Y231, and *Y. rochesterensis* ATCC 33639.

### Analysis of 16S rRNA gene variants in non-pathogenic *Yersinia* species

The sequences of 16S rRNA genes within the species were also analyzed according to species identification using core SNPs and ANI values. Table 5 shows the minimum and average 16S rRNA homology values and, the number of sequence variants within the *Yersinia* species. For each species, the most common 16S rRNA sequence was determined.

**Table 5:** Variability of 16S rRNA genes within non-pathogenic *Yersinia* species.

Species	Total quantity	Number of variants	The smallest homology, %	Average homology, %	The most common variant	
					Number	% of total quantity
*Y. ruckeri*	203	18	99.08	99.94	170	83.7
*Y. intermedia*	65	15	99.35	99.77	14	21.5
*Y. kristensenii*	47	11	99.08	99.59	17	36.2
*Y. massiliensis*	40	8	99.48	99.85	21	52.5
*Y. rochesterensis*	38	9	99.69	99.97	24	63.2
*Y. mollaretii*	32	9	99.67	99.93	12	37.5
*Y. frederiksenii*	28	7	99.61	99.91	13	46.4
*Y. alsatica*	22	12	99.28	99.71	8	36.4
*Y. bercovieri*	21	8	99.48	99.81	7^1^	33.3
*Y. rohdei*	18	6	99.67	99.92	12	66.7
*Y. aleksiciae*	17	5	99.81	99.94	10	58.8
*Y. similis*	15	2	99.94	99.99	14	93.3
*Y. aldovae*	14	12	98.43	99.42	2^1^	14.3
*Y. proxima*	11	4	99.61	99.87	5	45.5
*Y. canariae*	9	4	99.67	99.84	3^1^	33.3
*Y. hibernica*	9	2	99.87	99.97	8	88.9
*Y. vastinensis*	9	5	99.67	99.84	3	33.3
*Y. entomophaga*	7	5	99.54	99.86	4	57.1
*Y. artesiana*	4	1	100	100	4	100.0
*Y. thracica*	4	2	99.81	99.9	3	75.0
*Y. pekkanenii*	2	1	100	100	2	100
*Y. nurmii*	1	1		–	1	100

1 Two the same dominant variants of 16S rRNA gene.

The average 16S rRNA homology inside non-pathogenic *Yersinia* species exceeded 99%. The number of 16S rRNA variants in the species is likely depends on the total number of gene sequences. The largest number of 16S rRNA variants was 18 in *Y. ruckeri* (total 203 sequences). However, in *Y. aldovae*, 12 gene variants were identified among 14 sequences, and this species also has the smallest gene homology of 98.43%. 15 gene variants were found in *Y. intermedia*, but three of them are predominant, one of them observed in 14 cases, the second in 12, and the third 11. Two predominant sequences of *Y. mollaretii* were determined. They were found in 12 and 11 cases, respectively. Two dominant sequences were also found in *Y. bercovieri*, and both were observed in seven cases.

## Discussion

Since the 1980s, the 16S rRNA gene has been used in phylogenetic studies of bacteria (Clarridge, 2004). This gene has been considered as the best target for identification because it exists in all known prokaryotic genomes and has conserved and variable regions (Srinivasan et al., 2015). These properties made the 16S rRNA gene suitable for taxonomy studies. However, the presence of multiple copies of rRNA operons and intragenomic heterogenicity of 16S rRNA genes are the limiting factors for species identification (Muhamad Rizal et al., 2020; Watts et al., 2017). In the study were analyzed 2013 complete genomes of bacteria and archaea, and intragenomic heterogenicity was found in 952 genomes (585 species), but the divergence was less than 1% in 87.5% of the genomes (Sun et al., 2013).

Although the genus *Yersinia* contains three known human pathogens (*Y. pestis*, *Y. enterocolitica* and *Y. pseudotuberculosis*), the remaining species have not been studied sufficiently. Recently, with the expansion of genome studies, the taxonomy of the *Yersinia* genus has been continuously refined. In previous years, a few new species of *Yersinia* have been described. The genetic homology of 16S rRNA genes in the genus *Yersinia* is high, and even identical 16S rRNA gene sequences can be found between distinct species (Clarridge, 2004; Rodriguez-R et al., 2018). However, studies of 16S rRNA genes sequences, and using this gene for the identification of non-pathogenic *Yersinia* with verification by methods based on whole genome analysis are lacking. Hao et al. studied the identification of *Yersinia* spp. using copy diversity in the chromosomal 16S rRNA gene sequence. In this study, we used complete and draft genomes deposed from the NCBI Genome database, which is used by researchers worldwide. We analyzed the 16S rRNA genes and used ANI and core SNPs to identify the species of *Yersinia*. First, we compared the sequences of 16S rRNA gene copies in the complete genomes of non-human-pathogenic *Yersinia* from NCBI. The homology of 16S rRNA copies in each genome exceeded 99.5% for all strains except for one. It has been shown that many species have gene copies in their genomes that differ by 1–1.3% (Sun et al., 2013). The intragenomic heterogenicity of the 16S rRNA gene in complete *Yersinia* genomes is above the threshold for species determination and should not affect species identification. The intragenomic heterogenicity of the species *Y. wautersii*, *Y. vastinensis*, *Y. artesiana*, *Y. proxima*, *Y. pekkanenii*, *Y. thracica*, and *Y. nurmii* is unknown due to the absence of complete genomes available from the NCBI database. Only one complete genome of *Y. aldovae*, *Y. aleksiciae*, *Y. alsatica*, *Y. entomophaga*, *Y. kristensenii*, *Y. mollaretii*, *Y. rohdei*, and *Y. similis* was available; other non-pathogenic species were presented with only a few complete genomes. However, in a small cohort of *Yersinia*, above 50% of genomes have four or more variants of the 16S rRNA gene. It was revealed that among 327 investigated draft genomes of non-pathogenic *Yersinia*, 11% did not have a full-length 16S rRNA gene. Generally, the gene consists of two separate parts or is not assembled at 100% length. One copy of 16S rRNA contains 287 (87.8%) draft genomes. Typically generated by WGS short reads (100–600 bp) related to repeat regions of the genome are assembled into one variant. Generally, the algorithm used for assembly software eliminates variable nucleotides of lower frequency. In this case, it was impossible to evaluate the intragenomic heterogenicity of the 16S rRNA gene because it was compiled from single reads from seven probable different copies.

The investigations of 16S rRNA revealed that homology of genes belonging to different bacterial species is very high (Hao et al., 2016). In our study, the average degree of gene homology in non-pathogenic *Yersinia* was 98.76%, and the maximum variability was 2.85%. This high homology could limit the use of 16S rRNA for species identification of these bacteria. The low degree of genetic heterogenicity of the gene (0.36%) was determined in group *Y. pekkanenii*/*Y. proxima*/*Y. aldovae*/*Y. intermedia*/*Y. kristensenii*/*Y. rochesterensis*. Previously, it was reported that some bacterial species have identical 16S rRNA sequences (Rodriguez-R et al., 2018; Hao et al., 2016). In our study, identical gene sequences were found in the genomes of the *Y. intermedia* and *Y. rochesterensis* strains identified using ANI and core SNPs analyses.

16S rRNA genes are considered as species specific markers for prokaryotes phylogenetic studies. The studies have confirmed the existence of 16S rRNA horizontal transfer between different species, as well as intergenomic and intragenomic recombination of 16S rRNA gene regions (Kitahara and Miyazaki, 2013; Sun et al., 2013). All these facts indicate that using only the 16S rRNA gene for identification or phylogenetic studies is not rational.

In our study, the phylogenetic tree based on 16S rRNA genes differed from the tree based on core SNPs of the genomes. The phylogenetic analysis based on core SNPs showed that three species, *Y. ruckeri*, *Y. entomophaga*, and *Y. nurmii*, form a separate clade of the genus *Yersinia*, which is consistent with other studies. *Y. ruckeri* clusters with part of the genomes of *Y. kristensenii*, whereas *Y. entomophaga* and *Y. nurmii* are grouped with *Y. vastinensis* on the 16S rRNA phylogenetic tree. The species *Y. vastinensis* was recently described and genetically related to *Y. frederiksenii* and *Y. alsatica*. However, these two species are in another group, with *Y. pseudotuberculosis*, *Y. pestis*, *Y. similis*, and *Y. wautersii*, according to the 16S rRNA-based phylogenetic tree. Based on the results of core SNPs and ANI analyses, the *Y. pseudotuberculosis* complex and *Y. similis* form a separate clade of the genus *Yersinia*, which is not related to other species.

The species *Y. aldovae*, *Y. intermedia*, *Y. kristensenii*, *Y. rochesterensis*, *Y. pekkanenii*, and *Y. proxima* have 99.64% homology with 16S rRNA genes and are grouped together in the tree. On the phylogenetic tree based on core SNPs, the species *Y. aldovae*, *Y. intermedia*, and *Y. pekkanenii* form a clade consisting of separated branches corresponding to each species. The closely related species *Y. kristensenii*, *Y. thracica*, and *Y. rochesterensis* were grouped together; *Y. proxima* was located with *Y. enterocolitica* and its related species, *Y. artesiana* and *Y. canariae*.

The 16S rRNA gene phylogenetic tree, shows that the *Y. kristensenii* strains are separated into two groups. The first group forms a separate branch within group 1a, and the second group is included in the third group of 1b, consisting of high homologous 16S rRNA sequences of *Y. pekkanenii*, *Y. proxima*, *Y. aldovae*, *Y. intermedia*, *Y. kristensenii*, and *Y. rochesterensis*. The 16S rRNA gene sequences of strains *Y. rohdei* 68/02 and *Y. rohdei* 3,343 are clustered separately from other gene sequences of *Y. rohdei* strains that are grouped with *Y. thracica*.

In addition, in the phylogenetic tree based on the 16S rRNA sequences, some gene copies from complete genomes are located very far from each other because of high variability. Six gene copies of *Y. frederiksenii* Y225 were clustered into group 1b, and the seventh into group 3a. Six gene copies of *Y. aldovae* IP07632 were clustered into group 1b, and one into group 3b.

In our study, species identification of non-pathogenic *Yersinia* using core SNPs was correlated with ANI results. These methods are based on whole genome comparisons and allow to determine species identification and the relationship between strains. For species identification of poorly studied bacteria or those with high 16S rRNA gene homology, such as non-pathogenic *Yersinia*, it is better to use a set of methods based on whole genome analysis.

WGS provides information about the whole genome, and in addition to species identification, it can be used to study phylogeny and, identify resistance genes and, virulence factors, as well as plasmids, prophages, and other significant genetic traits.

Although the 16S rRNA gene is not well suited for studying phylogenetic relationships, this method compared with WGS is not expensive, performs easier, and does not require special technical staff. Nevertheless, extensive experience has been accumulated in using 16S rRNA genes to determine species identity, and convenient databases exist for interpreting the data. Because of the presence of this gene in all known microorganisms and the existence of universal primers, the 16S rRNA gene is a suitable target for studying metagenomic communities.

In summary, the 16S rRNA gene is not the most appropriate candidate gene for the accurate identification of *Yersinia* species. This is due to several reasons. The first limitation is the poorly studied non-pathogenic *Yersinia* and not enough numbers of genomes or sequences of 16S rRNA genes in the available databases. Second, in some cases, the results are difficult to interpret due to small differences between the 16S rRNA genes of the *Yersinia* genus (Clarridge, 2004). In our study, the average degree of gene homology was 98.76%. In some cases, identical 16S rRNA gene sequences correspond to different species (Rodriguez-R et al., 2018); in others, the distinction could be only one or a few nucleotides located outside the examined part of the gene. In addition, existing slightly different 16S rRNA gene variants within the species could be confused the researchers; especially if the species have limited available sequences or genomes in databases. The intragenomic heterogenicity of the 16S rRNA gene in complete *Yersinia* genomes exceeded the threshold for species determination and should not affect species identification. However, due to the small number of complete genomes in NCBI, this could not be clarified properly.

However, in a small cohort of *Yersinia* complete genomes, above 50% of them have four or more variants of the 16S rRNA gene in each. In most of the investigated draft genomes (87.8%), only one copy of 16S rRNA gene was present. The most likely this sequence was compiled from different copies of the gene. Besides this the full-length assembled 16S rRNA gene was absent in 36 drafts (9%). In additional, the other identification mismatches could be related to the development of the genus *Yersinia* taxonomy when there is no time to change old species names or revise data.

In our case, we determined that it was better to use core SNPs or ANI for accurate species identification of *Yersinia* strains than to sequence the 16S rRNA gene. Using for this purpose the methods based on whole genome comparison let to avoid misidentification. Of course, performing of whole genome sequencing and bioinformatics analysis requires expensive equipment and professionals, but in ambiguous and controversial cases, this is the best method for species identification and determining the phylogenetic relationship among strains.

## Data Availability

The datasets presented in this study can be found in online repositories. The names of the repository/repositories and accession number(s) can be found in the article/Supplementary material.

## Footnotes

^1^ https://github.com/tseemann/snippy

^2^ http://tree.bio.ed.ac.uk/software/figtree

^3^ https://github.com/husonlab/splitstree4

## Supplementary material

SUPPLEMENTARY TABLE S1 The complete and draft genomes of *Yersinia* used in the study.

SUPPLEMENTARY TABLE S2 Sequencing technologies, assembly methods and NCBI Genome accession numbers of *Yersinia* complete genomes used in the study.

SUPPLEMENTARY TABLE S3 Pairwise comparisons of the 16S rRNA genes of the *Yersinia* genomes used in the study.

SUPPLEMENTARY TABLE S4 ANI pairwise for the *Yersinia* genomes used in the study.

SUPPLEMENTARY FIGURE S1 Neighbor-joining phylogenetic tree based on 644 aligned genes of the 16S rRNA genus *Yersinia*.

SUPPLEMENTARY FIGURE S2 Neighbor-joining phylogenetic tree based on 644 aligned genes of the 16S rRNA genus *Yersinia*. The branches composed of identical genes or genes with some nucleotide differences were compressed.

SUPPLEMENTARY FIGURE S3 Neighbor joining tree based on 9494 core SNPs in *Yersinia* genomes. This tree was rooted using midpoint option. The groups of genomes observed in the tree were identified according to the species names of the type strains.
