# Supplementary material for: Evaluation of 16S rRNA genes sequences and genome-based analysis for identification of non-pathogenic Yersinia
Source: Front Microbiol. 2025 Jan 7;15:1519733. doi: 10.3389/fmicb.2024.1519733 (PMC11753223; doi:10.3389/fmicb.2024.1519733)
Supplement: SUPPLEMENTARY TABLE S1 — The complete and draft genomes of Yersinia used in the study. [file Table_1.docx]

Supplementary Table S1 The complete and draft genomes of *Yersinia* used in the study

|  | Species | Strain name | Assembly  accession | NCBI RefSeq assembly | Species identification by core SNPs and ANI |
| --- | --- | --- | --- | --- | --- |
|  | [*Y. frederiksenii*](https://www.ncbi.nlm.nih.gov/genome/873?genome_assembly_id=343758) | [FDAARGOS_417](https://www.ncbi.nlm.nih.gov/genome/873?genome_assembly_id=343758) | [CP023962](https://www.ncbi.nlm.nih.gov/nuccore/CP023962.1) | GCA_002591095.1 | *Y. massiliensis* |
|  | [*Y. frederiksenii*](https://www.ncbi.nlm.nih.gov/genome/873?genome_assembly_id=343758) | [FDAARGOS_418](https://www.ncbi.nlm.nih.gov/genome/873?genome_assembly_id=343759) | [CP023964](https://www.ncbi.nlm.nih.gov/nuccore/CP023964.1) | GCA_002591195.1 | [*Y. frederiksenii*](https://www.ncbi.nlm.nih.gov/genome/873?genome_assembly_id=343758) |
|  | [*Y. frederiksenii*](https://www.ncbi.nlm.nih.gov/genome/873?genome_assembly_id=343758) | [NCTC11470](https://www.ncbi.nlm.nih.gov/genome/873?genome_assembly_id=392057) | [UHJA01](https://www.ncbi.nlm.nih.gov/Traces/wgs/?val=UHJA01) | GCA_900460425.1 | [*Y. frederiksenii*](https://www.ncbi.nlm.nih.gov/genome/873?genome_assembly_id=343758) |
|  | [*Y. frederiksenii*](https://www.ncbi.nlm.nih.gov/genome/873?genome_assembly_id=343758) | [FE80151](https://www.ncbi.nlm.nih.gov/genome/873?genome_assembly_id=239503) | [CPVN01](https://www.ncbi.nlm.nih.gov/Traces/wgs/?val=CPVN01) | GCA_001158365.1 | [*Y. frederiksenii*](https://www.ncbi.nlm.nih.gov/genome/873?genome_assembly_id=343758) |
|  | [*Y. frederiksenii*](https://www.ncbi.nlm.nih.gov/genome/873?genome_assembly_id=343758) | [FE80258](https://www.ncbi.nlm.nih.gov/genome/873?genome_assembly_id=239492) | [CPWN01](https://www.ncbi.nlm.nih.gov/Traces/wgs/?val=CPWN01) | GCA_001098625.1 | *Y. alsatica* |
|  | [*Y. frederiksenii*](https://www.ncbi.nlm.nih.gov/genome/873?genome_assembly_id=343758) | [MGYG-HGUT-02467](https://www.ncbi.nlm.nih.gov/genome/873?genome_assembly_id=656840) | [CABMMI01](https://www.ncbi.nlm.nih.gov/Traces/wgs/?val=CABMMI01) | GCA_902387415.1 | *Y. alsatica* |
|  | [*Y. frederiksenii*](https://www.ncbi.nlm.nih.gov/genome/873?genome_assembly_id=343758) | [FE80988](https://www.ncbi.nlm.nih.gov/genome/873?genome_assembly_id=239495) | [CQEN01](https://www.ncbi.nlm.nih.gov/Traces/wgs/?val=CQEN01) | GCA_001135485.1 | *Y. massiliensis* |
|  | [*Y. frederiksenii*](https://www.ncbi.nlm.nih.gov/genome/873?genome_assembly_id=343758) | [3430](https://www.ncbi.nlm.nih.gov/genome/873?genome_assembly_id=239501) | [CQDU01](https://www.ncbi.nlm.nih.gov/Traces/wgs/?val=CQDU01) | GCA_001155565.1 | *Y. vastinensis* |
|  | [*Y. frederiksenii*](https://www.ncbi.nlm.nih.gov/genome/873?genome_assembly_id=343758) | [FCF208](https://www.ncbi.nlm.nih.gov/genome/873?genome_assembly_id=239493) | [CQBX01](https://www.ncbi.nlm.nih.gov/Traces/wgs/?val=CQBX01) | GCA_001105765.1 | *Y. vastinensis* |
|  | [*Y. frederiksenii*](https://www.ncbi.nlm.nih.gov/genome/873?genome_assembly_id=343758) | [CFSAN060534](https://www.ncbi.nlm.nih.gov/genome/873?genome_assembly_id=321078) | [NHOK01](https://www.ncbi.nlm.nih.gov/Traces/wgs/?val=NHOK01) | GCA_002188975.1 | *Y. alsatica* |
|  | [*Y. frederiksenii*](https://www.ncbi.nlm.nih.gov/genome/873?genome_assembly_id=343758) | [CFSAN060535](https://www.ncbi.nlm.nih.gov/genome/873?genome_assembly_id=321077) | [NHOJ01](https://www.ncbi.nlm.nih.gov/Traces/wgs/?val=NHOJ01) | GCA_002188925.1 | *Y. vastinensis* |
|  | [*Y. frederiksenii*](https://www.ncbi.nlm.nih.gov/genome/873?genome_assembly_id=343758) | [28/85](https://www.ncbi.nlm.nih.gov/genome/873?genome_assembly_id=239502) | [CQEP01](https://www.ncbi.nlm.nih.gov/Traces/wgs/?val=CQEP01) | GCA_001157485.1 | *Y. alsatica* |
|  | [*Y. frederiksenii*](https://www.ncbi.nlm.nih.gov/genome/873?genome_assembly_id=343758) | [38/83](https://www.ncbi.nlm.nih.gov/genome/873?genome_assembly_id=253695) | [CGCC01](https://www.ncbi.nlm.nih.gov/Traces/wgs/?val=CGCC01) | GCA_001319585.1 | [*Y. frederiksenii*](https://www.ncbi.nlm.nih.gov/genome/873?genome_assembly_id=343758) |
|  | [*Y. frederiksenii*](https://www.ncbi.nlm.nih.gov/genome/873?genome_assembly_id=343758) | [MGYG-HGUT-02466](https://www.ncbi.nlm.nih.gov/genome/873?genome_assembly_id=656841) | [CABMMH01](https://www.ncbi.nlm.nih.gov/Traces/wgs/?val=CABMMH01) | GCA_902387485.1 | [*Y. frederiksenii*](https://www.ncbi.nlm.nih.gov/genome/873?genome_assembly_id=343758) |
|  | [*Y. frederiksenii*](https://www.ncbi.nlm.nih.gov/genome/873?genome_assembly_id=343758) | [3317/84](https://www.ncbi.nlm.nih.gov/genome/873?genome_assembly_id=239496) | [CPYY01](https://www.ncbi.nlm.nih.gov/Traces/wgs/?val=CPYY01) | GCA_001142745.1 | [*Y. frederiksenii*](https://www.ncbi.nlm.nih.gov/genome/873?genome_assembly_id=343758) |
|  | [*Y. frederiksenii*](https://www.ncbi.nlm.nih.gov/genome/873?genome_assembly_id=343758) | [498/85](https://www.ncbi.nlm.nih.gov/genome/873?genome_assembly_id=239490) | [CQEC01](https://www.ncbi.nlm.nih.gov/Traces/wgs/?val=CQEC01) | GCA_001089645.1 | *Y. alsatica* |
|  | [*Y. frederiksenii*](https://www.ncbi.nlm.nih.gov/genome/873?genome_assembly_id=343758) | [IP25924](https://www.ncbi.nlm.nih.gov/genome/873?genome_assembly_id=239494) | [CQBF01](https://www.ncbi.nlm.nih.gov/Traces/wgs/?val=CQBF01) | GCA_001121225.1 | *Y. alsatica* |
|  | [*Y. frederiksenii*](https://www.ncbi.nlm.nih.gov/genome/873?genome_assembly_id=343758) | [22714/85](https://www.ncbi.nlm.nih.gov/genome/873?genome_assembly_id=239491) | [CQBO01](https://www.ncbi.nlm.nih.gov/Traces/wgs/?val=CQBO01) | GCA_001090165.1 | *Y. alsatica* |
|  | [*Y. frederiksenii*](https://www.ncbi.nlm.nih.gov/genome/873?genome_assembly_id=343758) | [FCF224](https://www.ncbi.nlm.nih.gov/genome/873?genome_assembly_id=239497) | [CQBW01](https://www.ncbi.nlm.nih.gov/Traces/wgs/?val=CQBW01) | GCA_001147045.1 | *Y. vastinensis* |
|  | [*Y. frederiksenii*](https://www.ncbi.nlm.nih.gov/genome/873?genome_assembly_id=343758) | [FCF343](https://www.ncbi.nlm.nih.gov/genome/873?genome_assembly_id=239498) | [CQBR01](https://www.ncbi.nlm.nih.gov/Traces/wgs/?val=CQBR01) | GCA_001147425.1 | *Y. alsatica* |
|  | [*Y. frederiksenii*](https://www.ncbi.nlm.nih.gov/genome/873?genome_assembly_id=343758) | [FCF467](https://www.ncbi.nlm.nih.gov/genome/873?genome_assembly_id=253696) | [CGCI01](https://www.ncbi.nlm.nih.gov/Traces/wgs/?val=CGCI01) | GCA_001319985.1 | *Y. massiliensis* |
|  | [*Y. frederiksenii*](https://www.ncbi.nlm.nih.gov/genome/873?genome_assembly_id=343758) | [IP37603](https://www.ncbi.nlm.nih.gov/genome/873?genome_assembly_id=647902) | [CABHXB01](https://www.ncbi.nlm.nih.gov/Traces/wgs/?val=CABHXB01) | GCA_902170235.1 | [*Y. frederiksenii*](https://www.ncbi.nlm.nih.gov/genome/873?genome_assembly_id=343758) |
|  | [*Y. frederiksenii*](https://www.ncbi.nlm.nih.gov/genome/873?genome_assembly_id=343758) | [RS-42](https://www.ncbi.nlm.nih.gov/genome/873?genome_assembly_id=240488) | [CQDT01](https://www.ncbi.nlm.nih.gov/Traces/wgs/?val=CQDT01) | GCA_001171065.1 | *Y. alsatica* |
|  | [*Y. frederiksenii*](https://www.ncbi.nlm.nih.gov/genome/873?genome_assembly_id=343758) | [IP23047](https://www.ncbi.nlm.nih.gov/genome/873?genome_assembly_id=253693) | [CGBN01](https://www.ncbi.nlm.nih.gov/Traces/wgs/?val=CGBN01) | GCA_001319525.1 | *Y. alsatica* |
|  | [*Y. frederiksenii*](https://www.ncbi.nlm.nih.gov/genome/873?genome_assembly_id=343758) | [IP23698](https://www.ncbi.nlm.nih.gov/genome/873?genome_assembly_id=239500) | [CPZP01](https://www.ncbi.nlm.nih.gov/Traces/wgs/?val=CPZP01) | GCA_001151185.1 | *Y. massiliensis* |
|  | [*Y. frederiksenii*](https://www.ncbi.nlm.nih.gov/genome/873?genome_assembly_id=343758) | [112/02](https://www.ncbi.nlm.nih.gov/genome/873?genome_assembly_id=244210) | [CTKH01](https://www.ncbi.nlm.nih.gov/Traces/wgs/?val=CTKH01) | GCA_001222845.1 | *Y. massiliensis* |
|  | [*Y. frederiksenii*](https://www.ncbi.nlm.nih.gov/genome/873?genome_assembly_id=343758) | [3400/83](https://www.ncbi.nlm.nih.gov/genome/873?genome_assembly_id=253694) | [CGCB01](https://www.ncbi.nlm.nih.gov/Traces/wgs/?val=CGCB01) | GCA_001319565.1 | *Y. massiliensis* |
|  | [*Y. frederiksenii*](https://www.ncbi.nlm.nih.gov/genome/873?genome_assembly_id=343758) | [MGYG-HGUT-02465](https://www.ncbi.nlm.nih.gov/genome/873?genome_assembly_id=656842) | [CABMMF01](https://www.ncbi.nlm.nih.gov/Traces/wgs/?val=CABMMF01) | GCA_902387415.1 | *Y. massiliensis* |
|  | [*Y. frederiksenii*](https://www.ncbi.nlm.nih.gov/genome/873?genome_assembly_id=343758) | [120/02](https://www.ncbi.nlm.nih.gov/genome/873?genome_assembly_id=244209) | [CTJA01](https://www.ncbi.nlm.nih.gov/Traces/wgs/?val=CTJA01) | GCA_001218185.1 | *Y. massiliensis* |
|  | [*Y. frederiksenii*](https://www.ncbi.nlm.nih.gov/genome/873?genome_assembly_id=343758) | [IP38678](https://www.ncbi.nlm.nih.gov/genome/873?genome_assembly_id=647916) | [CABHYA01](https://www.ncbi.nlm.nih.gov/Traces/wgs/?val=CABHYA01) | GCA_902170525.1 | [*Y. frederiksenii*](https://www.ncbi.nlm.nih.gov/genome/873?genome_assembly_id=343758) |
|  | [*Y. frederiksenii*](https://www.ncbi.nlm.nih.gov/genome/873?genome_assembly_id=343758) | [IP39768](https://www.ncbi.nlm.nih.gov/genome/873?genome_assembly_id=647917) | [CABHXP01](https://www.ncbi.nlm.nih.gov/Traces/wgs/?val=CABHXP01) | GCA_902170545.1 | [*Y. frederiksenii*](https://www.ncbi.nlm.nih.gov/genome/873?genome_assembly_id=343758) |
|  | [*Y. frederiksenii*](https://www.ncbi.nlm.nih.gov/genome/873?genome_assembly_id=343758) | [ATCC 33641](https://www.ncbi.nlm.nih.gov/genome/873?genome_assembly_id=208415) | [JPPS01](https://www.ncbi.nlm.nih.gov/Traces/wgs/?val=JPPS01) | GCA_000754805.1 | [*Y. frederiksenii*](https://www.ncbi.nlm.nih.gov/genome/873?genome_assembly_id=343758) |
|  | [*Y. frederiksenii*](https://www.ncbi.nlm.nih.gov/genome/873?genome_assembly_id=343758) | [ATCC 33641](https://www.ncbi.nlm.nih.gov/genome/873?genome_assembly_id=169812) | [AALE02](https://www.ncbi.nlm.nih.gov/Traces/wgs/?val=AALE02) | GCA_000168015.1 | [*Y. frederiksenii*](https://www.ncbi.nlm.nih.gov/genome/873?genome_assembly_id=343758) |
|  | [*Y. frederiksenii*](https://www.ncbi.nlm.nih.gov/genome/873?genome_assembly_id=343758) | [SCPM-O-B-3986](https://www.ncbi.nlm.nih.gov/genome/873?genome_assembly_id=321578) | [MWTK01](https://www.ncbi.nlm.nih.gov/Traces/wgs/?val=MWTK01) | GCA_002192925.1 | *Y. massiliensis* |
|  | [*Y. frederiksenii*](https://www.ncbi.nlm.nih.gov/genome/873?genome_assembly_id=343758) | [SCPM-O-B-7604](https://www.ncbi.nlm.nih.gov/genome/873?genome_assembly_id=321576) | [MWTI01](https://www.ncbi.nlm.nih.gov/Traces/wgs/?val=MWTI01) | GCA_002192815.1 | *Y. alsatica* |
|  | [*Y. frederiksenii*](https://www.ncbi.nlm.nih.gov/genome/873?genome_assembly_id=343758) | [SCPM-O-B-8031](https://www.ncbi.nlm.nih.gov/genome/873?genome_assembly_id=321577) | [MWTJ01](https://www.ncbi.nlm.nih.gov/Traces/wgs/?val=MWTJ01) | GCA_002192835.1 | *Y. alsatica* |
|  | [*Y. frederiksenii*](https://www.ncbi.nlm.nih.gov/genome/873?genome_assembly_id=343758) | [Y225](https://www.ncbi.nlm.nih.gov/genome/873?genome_assembly_id=219471) | [CP009364](https://www.ncbi.nlm.nih.gov/nuccore/CP009364.1) | GCA_000834215.1 | *Y. rochesterensis* |
|  | *Y. bercovieri* | [IZSPB_Y87](https://www.ncbi.nlm.nih.gov/genome/1689?genome_assembly_id=1723508) | [JAJAVX01](https://www.ncbi.nlm.nih.gov/Traces/wgs/?val=JAJAVX01) | GCA_020531725.1 | *Y. bercovieri* |
|  | *Y. bercovieri* | [127/84](https://www.ncbi.nlm.nih.gov/genome/1689?genome_assembly_id=239566) | [CQBU01](https://www.ncbi.nlm.nih.gov/Traces/wgs/?val=CQBU01) | GCA_001131685.1 | *Y. bercovieri* |
|  | *Y. bercovieri* | [FE80217](https://www.ncbi.nlm.nih.gov/genome/1689?genome_assembly_id=964674) | [CGBH01](https://www.ncbi.nlm.nih.gov/Traces/wgs/?val=CGBH01) | GCA_001319955.1 | *Y. bercovieri* |
|  | *Y. bercovieri* | [MGYG-HGUT-02524](https://www.ncbi.nlm.nih.gov/genome/1689?genome_assembly_id=656937) | [CABMOD01](https://www.ncbi.nlm.nih.gov/Traces/wgs/?val=CABMOD01) | GCA_902388035.1 | *Y. bercovieri* |
|  | *Y. bercovieri* | [3016/84](https://www.ncbi.nlm.nih.gov/genome/1689?genome_assembly_id=253909) | [CHYZ01](https://www.ncbi.nlm.nih.gov/Traces/wgs/?val=CHYZ01) | GCA_001319545.1 | *Y. bercovieri* |
|  | *Y. bercovieri* | [IP39738](https://www.ncbi.nlm.nih.gov/genome/1689?genome_assembly_id=648377) | [CABHPT01](https://www.ncbi.nlm.nih.gov/Traces/wgs/?val=CABHPT01) | GCA_902168565.1 | *Y. bercovieri* |
|  | *Y. bercovieri* | [IP26086](https://www.ncbi.nlm.nih.gov/genome/1689?genome_assembly_id=648372) | [CABHPW01](https://www.ncbi.nlm.nih.gov/Traces/wgs/?val=CABHPW01) | GCA_902168485.1 | *Y. bercovieri* |
|  | *Y. bercovieri* | [IP36520](https://www.ncbi.nlm.nih.gov/genome/1689?genome_assembly_id=648375) | [CABHQB01](https://www.ncbi.nlm.nih.gov/Traces/wgs/?val=CABHQB01) | GCA_902168545.1 | *Y. bercovieri* |
|  | *Y. bercovieri* | [IP37104](https://www.ncbi.nlm.nih.gov/genome/1689?genome_assembly_id=648379) | [CABHQJ01](https://www.ncbi.nlm.nih.gov/Traces/wgs/?val=CABHQJ01) | GCA_902168635.1 | *Y. bercovieri* |
|  | *Y. bercovieri* | [IP27502](https://www.ncbi.nlm.nih.gov/genome/1689?genome_assembly_id=648376) | [CABHPV01](https://www.ncbi.nlm.nih.gov/Traces/wgs/?val=CABHPV01) | GCA_902168555.1 | *Y. bercovieri* |
|  | *Y. bercovieri* | [IP36824](https://www.ncbi.nlm.nih.gov/genome/1689?genome_assembly_id=648378) | [CABHQL01](https://www.ncbi.nlm.nih.gov/Traces/wgs/?val=CABHQL01) | GCA_902168625.1 | *Y. bercovieri* |
|  | *Y. bercovieri* | [IP25873](https://www.ncbi.nlm.nih.gov/genome/1689?genome_assembly_id=648373) | [CABHQH01](https://www.ncbi.nlm.nih.gov/Traces/wgs/?val=CABHQH01) | GCA_902168505.1 | *Y. bercovieri* |
|  | *Y. bercovieri* | [IP33686](https://www.ncbi.nlm.nih.gov/genome/1689?genome_assembly_id=648374) | [CABHQF01](https://www.ncbi.nlm.nih.gov/Traces/wgs/?val=CABHQF01) | GCA_902168535.1 | *Y. bercovieri* |
|  | *Y. bercovieri* | [IP38896](https://www.ncbi.nlm.nih.gov/genome/1689?genome_assembly_id=648380) | [CABHQM01](https://www.ncbi.nlm.nih.gov/Traces/wgs/?val=CABHQM01) | GCA_902168615.1 | *Y. bercovieri* |
|  | *Y. bercovieri* | [SCPM-O-B-7607](https://www.ncbi.nlm.nih.gov/genome/1689?genome_assembly_id=351821) | [PEHN01](https://www.ncbi.nlm.nih.gov/Traces/wgs/?val=PEHN01) | GCA_002738385.1 | *Y. bercovieri* |
|  | *Y. bercovieri* | [ATCC 43970](https://www.ncbi.nlm.nih.gov/genome/1689?genome_assembly_id=906995) | CP054044 | GCA_013282745.1 | *Y. bercovieri* |
|  | *Y. bercovieri* | [ATCC 43970](https://www.ncbi.nlm.nih.gov/genome/1689?genome_assembly_id=906995) | AALC02 | GCA_000167975.1 | *Y. bercovieri* |
|  | *Y. mollaretii* | [IP39060](https://www.ncbi.nlm.nih.gov/genome/1397?genome_assembly_id=648364) | [CABHYF01](https://www.ncbi.nlm.nih.gov/Traces/wgs/?val=CABHYF01) | GCA_902170755.1 | *Y. mollaretii* |
|  | *Y. mollaretii* | [FE82747](https://www.ncbi.nlm.nih.gov/genome/1397?genome_assembly_id=239558) | [CQBM01](https://www.ncbi.nlm.nih.gov/Traces/wgs/?val=CQBM01) | GCA_001127065.1 | *Y. mollaretii* |
|  | *Y. mollaretii* | [MGYG-HGUT-02471](https://www.ncbi.nlm.nih.gov/genome/1397?genome_assembly_id=656915) | [CABMMJ01](https://www.ncbi.nlm.nih.gov/Traces/wgs/?val=CABMMJ01) | GCA_902387525.1 | *Y. mollaretii* |
|  | *Y. mollaretii* | [IP27339](https://www.ncbi.nlm.nih.gov/genome/1397?genome_assembly_id=648359) | [CABHYS01](https://www.ncbi.nlm.nih.gov/Traces/wgs/?val=CABHYS01) | GCA_902170645.1 | *Y. mollaretii* |
|  | *Y. mollaretii* | [IP26765](https://www.ncbi.nlm.nih.gov/genome/1397?genome_assembly_id=648363) | [CABHYJ01](https://www.ncbi.nlm.nih.gov/Traces/wgs/?val=CABHYJ01) | GCA_902170745.1 | *Y. mollaretii* |
|  | *Y. mollaretii* | [61/02](https://www.ncbi.nlm.nih.gov/genome/1397?genome_assembly_id=244730) | [CTKJ01](https://www.ncbi.nlm.nih.gov/Traces/wgs/?val=CTKJ01) | GCA_001218645.1 | *Y. mollaretii* |
|  | *Y. mollaretii* | [IP28910](https://www.ncbi.nlm.nih.gov/genome/1397?genome_assembly_id=648360) | [CABHYK01](https://www.ncbi.nlm.nih.gov/Traces/wgs/?val=CABHYK01) | GCA_902170675.1 | *Y. mollaretii* |
|  | *Y. mollaretii* | [IP28240](https://www.ncbi.nlm.nih.gov/genome/1397?genome_assembly_id=648361) | [CABHYE01](https://www.ncbi.nlm.nih.gov/Traces/wgs/?val=CABHYE01) | GCA_902170695.1 | *Y. mollaretii* |
|  | *Y. mollaretii* | [IP36688](https://www.ncbi.nlm.nih.gov/genome/1397?genome_assembly_id=648358) | [CABHYV01](https://www.ncbi.nlm.nih.gov/Traces/wgs/?val=CABHYV01) | GCA_902170635.1 | *Y. mollaretii* |
|  | *Y. mollaretii* | [IP33651](https://www.ncbi.nlm.nih.gov/genome/1397?genome_assembly_id=648365) | [CABHYO01](https://www.ncbi.nlm.nih.gov/Traces/wgs/?val=CABHYO01) | GCA_902170795.1 | *Y. mollaretii* |
|  | *Y. mollaretii* | [107/02](https://www.ncbi.nlm.nih.gov/genome/1397?genome_assembly_id=244731) | [CTIO01](https://www.ncbi.nlm.nih.gov/Traces/wgs/?val=CTIO01) | GCA_001220765.1 | *Y. mollaretii* |
|  | *Y. mollaretii* | [IP36729](https://www.ncbi.nlm.nih.gov/genome/1397?genome_assembly_id=648362) | [CABHYM01](https://www.ncbi.nlm.nih.gov/Traces/wgs/?val=CABHYM01) | GCA_902170725.1 | *Y. mollaretii* |
|  | *Y. mollaretii* | [IP37695](https://www.ncbi.nlm.nih.gov/genome/1397?genome_assembly_id=648357) | [CABHYI01](https://www.ncbi.nlm.nih.gov/Traces/wgs/?val=CABHYI01) | GCA_902170625.1 | *Y. mollaretii* |
|  | *Y. mollaretii* | [92/84](https://www.ncbi.nlm.nih.gov/genome/1397?genome_assembly_id=253899) | [CIFJ01](https://www.ncbi.nlm.nih.gov/Traces/wgs/?val=CIFJ01) | GCA_001319605.1 | *Y. mollaretii* |
|  | *Y. mollaretii* | [64/02](https://www.ncbi.nlm.nih.gov/genome/1397?genome_assembly_id=244732) | [CTRC01](https://www.ncbi.nlm.nih.gov/Traces/wgs/?val=CTRC01) | GCA_001220845.1 | *Y. mollaretii* |
|  | *Y. mollaretii* | [IP25089](https://www.ncbi.nlm.nih.gov/genome/1397?genome_assembly_id=239559) | [CQDS01](https://www.ncbi.nlm.nih.gov/Traces/wgs/?val=CQDS01) | GCA_001149645.1 | *Y. mollaretii* |
|  | *Y. mollaretii* | [IP22404](https://www.ncbi.nlm.nih.gov/genome/1397?genome_assembly_id=239557) | [CPYJ01](https://www.ncbi.nlm.nih.gov/Traces/wgs/?val=CPYJ01) | GCA_001108385.1 | *Y. mollaretii* |
|  | *Y. mollaretii* | [58/02](https://www.ncbi.nlm.nih.gov/genome/1397?genome_assembly_id=244733) | [CTEP01](https://www.ncbi.nlm.nih.gov/Traces/wgs/?val=CTEP01) | GCA_001222925.1 | *Y. mollaretii* |
|  | *Y. mollaretii* | [SCPM-O-B-7596 (846/98)](https://www.ncbi.nlm.nih.gov/genome/1397?genome_assembly_id=839215) | [JAASAJ01](https://www.ncbi.nlm.nih.gov/Traces/wgs/?val=JAASAJ01) | GCA_011765205.1 | *Y. mollaretii* |
|  | *Y. mollaretii* | [SCPM-O-B-7598](https://www.ncbi.nlm.nih.gov/genome/1397?genome_assembly_id=351820) | [PEHO01](https://www.ncbi.nlm.nih.gov/Traces/wgs/?val=PEHO01) | GCA_002738475.1 | *Y. mollaretii* |
|  | *Y. mollaretii* | [SCPM-O-B-7609](https://www.ncbi.nlm.nih.gov/genome/1397?genome_assembly_id=354262) | [PGLU01](https://www.ncbi.nlm.nih.gov/Traces/wgs/?val=PGLU01) | GCA_002794235.1 | *Y. mollaretii* |
|  | *Y. mollaretii* | [SCPM-O-B-7610 (282/86)](https://www.ncbi.nlm.nih.gov/genome/1397?genome_assembly_id=839216) | [JAASAI01](https://www.ncbi.nlm.nih.gov/Traces/wgs/?val=JAASAI01) | GCA_011765325.1 | *Y. mollaretii* |
|  | *Y. mollaretii* | [ATCC 43969](https://www.ncbi.nlm.nih.gov/genome/1397?genome_assembly_id=906994) | CP054043 | GCA_000167995.1 | *Y. mollaretii* |
|  | *Y. mollaretii* | [ATCC 43969](https://www.ncbi.nlm.nih.gov/genome/1397?genome_assembly_id=171236) | [AALD02](https://www.ncbi.nlm.nih.gov/Traces/wgs/?val=AALD02) | GCA_013282725.1 | *Y. mollaretii* |
|  | *Y. mollaretii* | SCPM-O-B-7597 (H87/82) | JAQISH01 | GCF_027947235.1 | *Y. mollaretii* |
|  | *Y. mollaretii* | SCPM-O-B-8306 (333) | JAQIRS01 | GCF_027946935.1 | *Y. mollaretii* |
|  | *Y. mollaretii* | SCPM-O-B-10207 (176-36) | JAVKVJ01 | GCF_031462545.1 | *Y. mollaretii* |
|  | *Y. intermedia* | [IP10209](https://www.ncbi.nlm.nih.gov/genome/1398?genome_assembly_id=240737) | [CPVI01](https://www.ncbi.nlm.nih.gov/Traces/wgs/?val=CPVI01) | GCA_001167085.1 | *Y. intermedia* |
|  | *Y. intermedia* | [BR165/97](https://www.ncbi.nlm.nih.gov/genome/1398?genome_assembly_id=239560) | [CPZJ01](https://www.ncbi.nlm.nih.gov/Traces/wgs/?val=CPZJ01) | GCA_001088505.1 | *Y. intermedia* |
|  | *Y. intermedia* | [IP37946](https://www.ncbi.nlm.nih.gov/genome/1398?genome_assembly_id=648366) | [CABHXJ01](https://www.ncbi.nlm.nih.gov/Traces/wgs/?val=CABHXJ01) | GCA_902170445.1 | *Y. intermedia* |
|  | *Y. intermedia* | [IP10066](https://www.ncbi.nlm.nih.gov/genome/1398?genome_assembly_id=253900) | [CHZI01](https://www.ncbi.nlm.nih.gov/Traces/wgs/?val=CHZI01) | GCA_001319645.1 | *Y. intermedia* |
|  | *Y. intermedia* | [IP36595](https://www.ncbi.nlm.nih.gov/genome/1398?genome_assembly_id=648369) | [CABHXU01](https://www.ncbi.nlm.nih.gov/Traces/wgs/?val=CABHXU01) | GCA_902170555.1 | *Y. intermedia* |
|  | *Y. intermedia* | [93/02](https://www.ncbi.nlm.nih.gov/genome/1398?genome_assembly_id=244735) | [CTKY01](https://www.ncbi.nlm.nih.gov/Traces/wgs/?val=CTKY01) | GCA_001221165.1 | *Y. intermedia* |
|  | *Y. intermedia* | [821/84](https://www.ncbi.nlm.nih.gov/genome/1398?genome_assembly_id=240736) | [CQBB01](https://www.ncbi.nlm.nih.gov/Traces/wgs/?val=CQBB01) | GCA_001166625.1 | *Y. intermedia* |
|  | *Y. intermedia* | [FE80200](https://www.ncbi.nlm.nih.gov/genome/1398?genome_assembly_id=244738) | [CWJO01](https://www.ncbi.nlm.nih.gov/Traces/wgs/?val=CWJO01) | GCA_001244675.1 | *Y. intermedia* |
|  | *Y. intermedia* | [IZSPB_Y93](https://www.ncbi.nlm.nih.gov/genome/1398?genome_assembly_id=1723504) | [JAJAVY01](https://www.ncbi.nlm.nih.gov/Traces/wgs/?val=JAJAVY01) | GCA_020531695.1 | *Y. intermedia* |
|  | *Y. intermedia* | [IZSPB_Y97](https://www.ncbi.nlm.nih.gov/genome/1398?genome_assembly_id=1723505) | [JAJAVZ01](https://www.ncbi.nlm.nih.gov/Traces/wgs/?val=JAJAVZ01) | GCA_020531785.1 | *Y. intermedia* |
|  | *Y. intermedia* | [58735](https://www.ncbi.nlm.nih.gov/genome/1398?genome_assembly_id=239562) | [CQER01](https://www.ncbi.nlm.nih.gov/Traces/wgs/?val=CQER01) | GCA_001116865.1 | *Y. proxima* |
|  | *Y. intermedia* | [R148](https://www.ncbi.nlm.nih.gov/genome/1398?genome_assembly_id=244737) | [CWJI01](https://www.ncbi.nlm.nih.gov/Traces/wgs/?val=CWJI01) | GCA_001244565.1 | *Y. massiliensis* |
|  | *Y. intermedia* | [9/85](https://www.ncbi.nlm.nih.gov/genome/1398?genome_assembly_id=239565) | [CPWY01](https://www.ncbi.nlm.nih.gov/Traces/wgs/?val=CPWY01) | GCA_001157385.1 | *Y. intermedia* |
|  | *Y. intermedia* | [MGYG-HGUT-02525](https://www.ncbi.nlm.nih.gov/genome/1398?genome_assembly_id=656916) | [CABMNY01](https://www.ncbi.nlm.nih.gov/Traces/wgs/?val=CABMNY01) | GCA_902388045.1 | *Y. intermedia* |
|  | *Y. intermedia* | [FCF202](https://www.ncbi.nlm.nih.gov/genome/1398?genome_assembly_id=239564) | [CQBP01](https://www.ncbi.nlm.nih.gov/Traces/wgs/?val=CQBP01) | GCA_001153385. 1 | *Y. intermedia* |
|  | *Y. intermedia* | [FCF84](https://www.ncbi.nlm.nih.gov/genome/1398?genome_assembly_id=239563) | [CPVD01](https://www.ncbi.nlm.nih.gov/Traces/wgs/?val=CPVD01) | GCA_001140765.1 | *Y. intermedia* |
|  | *Y. intermedia* | [CFSAN060536](https://www.ncbi.nlm.nih.gov/genome/1398?genome_assembly_id=321087) | [NHOI01](https://www.ncbi.nlm.nih.gov/Traces/wgs/?val=NHOI01) | GCA_002188935.1 | *Y. intermedia* |
|  | *Y. intermedia* | [FCF335](https://www.ncbi.nlm.nih.gov/genome/1398?genome_assembly_id=239561) | [CQCT01](https://www.ncbi.nlm.nih.gov/Traces/wgs/?val=CQCT01) | GCA_001104645.1 | *Y. intermedia* |
|  | *Y. intermedia* | [CFSAN060537](https://www.ncbi.nlm.nih.gov/genome/1398?genome_assembly_id=321088) | [NHOH01](https://www.ncbi.nlm.nih.gov/Traces/wgs/?val=NHOH01) | GCA_002188995.1 | *Y. intermedia* |
|  | *Y. intermedia* | [IP38854](https://www.ncbi.nlm.nih.gov/genome/1398?genome_assembly_id=648368) | [CABHXW01](https://www.ncbi.nlm.nih.gov/Traces/wgs/?val=CABHXW01) | GCA_902170515.1 | *Y. intermedia* |
|  | *Y. intermedia* | [IP39994](https://www.ncbi.nlm.nih.gov/genome/1398?genome_assembly_id=648370) | [CABHYB01](https://www.ncbi.nlm.nih.gov/Traces/wgs/?val=CABHYB01) | GCA_902170585.1 | *Y. intermedia* |
|  | *Y. intermedia* | [FCF130](https://www.ncbi.nlm.nih.gov/genome/1398?genome_assembly_id=253901) | [CVJX01](https://www.ncbi.nlm.nih.gov/Traces/wgs/?val=CVJX01) | GCA_001319925.1 | *Y. intermedia* |
|  | *Y. intermedia* | [IZSPB_Y83](https://www.ncbi.nlm.nih.gov/genome/1398?genome_assembly_id=1723506) | [JAJAVW01](https://www.ncbi.nlm.nih.gov/Traces/wgs/?val=JAJAVW01) | GCA_020531805.1 | *Y. intermedia* |
|  | *Y. intermedia* | [IZSPB_Y82](https://www.ncbi.nlm.nih.gov/genome/1398?genome_assembly_id=1723507) | [JAJAVV01](https://www.ncbi.nlm.nih.gov/Traces/wgs/?val=JAJAVV01) | GCA_020531685.1 | *Y. intermedia* |
|  | *Y. intermedia* | [182/02](https://www.ncbi.nlm.nih.gov/genome/1398?genome_assembly_id=244734) | [CTFU01](https://www.ncbi.nlm.nih.gov/Traces/wgs/?val=CTFU01) | GCA_001218045.1 | *Y. intermedia* |
|  | *Y. intermedia* | [BR166/97](https://www.ncbi.nlm.nih.gov/genome/1398?genome_assembly_id=964666) | [CQBG01](https://www.ncbi.nlm.nih.gov/Traces/wgs/?val=CQBG01) | GCA_001150525.1 | *Y. intermedia* |
|  | *Y. intermedia* | [IP36321](https://www.ncbi.nlm.nih.gov/genome/1398?genome_assembly_id=648367) | [CABHXO01](https://www.ncbi.nlm.nih.gov/Traces/wgs/?val=CABHXO01) | GCA_902170485.1 | *Y. intermedia* |
|  | *Y. intermedia* | [ATCC 29909](https://www.ncbi.nlm.nih.gov/genome/1398?genome_assembly_id=171237) | [AALF02](https://www.ncbi.nlm.nih.gov/Traces/wgs/?val=AALF02) | GCA_000168035.1 | *Y. intermedia* |
|  | *Y. intermedia* | [SCPM-O-B-7605](https://www.ncbi.nlm.nih.gov/genome/1398?genome_assembly_id=321582) | [MWTO01](https://www.ncbi.nlm.nih.gov/Traces/wgs/?val=MWTO01) | GCA_002192885.1 | *Y. intermedia* |
|  | *Y. intermedia* | [NCTC11469](https://www.ncbi.nlm.nih.gov/genome/1398?genome_assembly_id=432428) | [LR134116](https://www.ncbi.nlm.nih.gov/nuccore/LR134116.1) | GCA_900635455.1 | *Y. intermedia* |
|  | *Y. intermedia* | [FDAARGOS_358](https://www.ncbi.nlm.nih.gov/genome/1398?genome_assembly_id=366596) | [CP027397](https://www.ncbi.nlm.nih.gov/nuccore/CP027397.1) | GCA_002983625.1 | *Y. intermedia* |
|  | *Y. intermedia* | [N6/293](https://www.ncbi.nlm.nih.gov/genome/1398?genome_assembly_id=1810975) | [CP093322](https://www.ncbi.nlm.nih.gov/nuccore/CP093322.1) | GCA_022637335.1 | *Y. intermedia* |
|  | *Y. intermedia* | [FDAARGOS_729](https://www.ncbi.nlm.nih.gov/genome/1398?genome_assembly_id=747694) | [CP046294](https://www.ncbi.nlm.nih.gov/nuccore/CP046294.1) | GCA_009730075.1 | *Y. intermedia* |
|  | *Y. intermedia* | [FDAARGOS_730](https://www.ncbi.nlm.nih.gov/genome/1398?genome_assembly_id=747695) | [CP046293](https://www.ncbi.nlm.nih.gov/nuccore/CP046293.1) | GCA_009730055.1 | *Y. intermedia* |
|  | *Y. intermedia* | [Y228](https://www.ncbi.nlm.nih.gov/genome/1398?genome_assembly_id=219478) | [CP009801](https://www.ncbi.nlm.nih.gov/nuccore/CP009801.1) | GCA_000834515.1 | *Y. intermedia* |
|  | *Y. intermedia* | SCPM-O-B-10209 (333) | JBHFPP01 | GCA_042128985.1 | *Y. intermedia* |
|  | *Y. intermedia* | SCPM-O-B-8114 (334) | JAQISA01 | GCA_027947095.1 | *Y. intermedia* |
|  | *Y. intermedia* | SCPM-O-B-8304 (335) | JAQIRZ01 | GCA_027947015.1 | *Y. intermedia* |
|  | *Y. intermedia* | SCPM-O-B-8305 (H357/85) | JAQIRY01 | GCA_027947055.1 | *Y. intermedia* |
|  | *Y. intermedia* | SCPM-O-B-3971 (680) | JAQIRX01 | GCA_027947025.1 | *Y. intermedia* |
|  | *Y. intermedia* | SCPM-O-B-10208 (116 INT) | JAPFIH01 | GCA_026183875.1 | *Y. intermedia* |
|  | *Y. rohdei* | [68/02](https://www.ncbi.nlm.nih.gov/genome/1758?genome_assembly_id=244740) | [CTKE01](https://www.ncbi.nlm.nih.gov/Traces/wgs/?val=CTKE01) | GCA_001217805.1 | *Y. rohdei* |
|  | *Y. rohdei* | [56/02](https://www.ncbi.nlm.nih.gov/genome/1758?genome_assembly_id=244741) | [CTKU01](https://www.ncbi.nlm.nih.gov/Traces/wgs/?val=CTKU01) | GCA_001223385.1 | *Y. rohdei* |
|  | *Y. rohdei* | [IP36459](https://www.ncbi.nlm.nih.gov/genome/1758?genome_assembly_id=648394) | [CABIHO01](https://www.ncbi.nlm.nih.gov/Traces/wgs/?val=CABIHO01) | GCA_902173205.1 | *Y. rohdei* |
|  | *Y. rohdei* | [IP38566](https://www.ncbi.nlm.nih.gov/genome/1758?genome_assembly_id=648392) | [CABIHY01](https://www.ncbi.nlm.nih.gov/Traces/wgs/?val=CABIHY01) | GCA_902173185.1 | *Y. rohdei* |
|  | *Y. rohdei* | [IP38305](https://www.ncbi.nlm.nih.gov/genome/1758?genome_assembly_id=648395) | [CABIHW01](https://www.ncbi.nlm.nih.gov/Traces/wgs/?val=CABIHW01) | GCA_902173225.1 | *Y. rohdei* |
|  | *Y. rohdei* | [IP38583](https://www.ncbi.nlm.nih.gov/genome/1758?genome_assembly_id=648393) | [CABIHQ01](https://www.ncbi.nlm.nih.gov/Traces/wgs/?val=CABIHQ01) | GCA_902173195.1 | *Y. rohdei* |
|  | *Y. rohdei* | [82589](https://www.ncbi.nlm.nih.gov/genome/1758?genome_assembly_id=239570) | [CQCB01](https://www.ncbi.nlm.nih.gov/Traces/wgs/?val=CQCB01) | GCA_001152185.1 | *Y. rohdei* |
|  | *Y. rohdei* | [YRA](https://www.ncbi.nlm.nih.gov/genome/1758?genome_assembly_id=219480) | [CP009787](https://www.ncbi.nlm.nih.gov/nuccore/CP009787.1) | GCA_000834455.1 | *Y. rohdei* |
|  | *Y. rohdei* | [3343](https://www.ncbi.nlm.nih.gov/genome/1758?genome_assembly_id=253910) | [CIFE01](https://www.ncbi.nlm.nih.gov/Traces/wgs/?val=CIFE01) | GCA_001320025.1 | *Y. rohdei* |
|  | *Y. rohdei* | [IP34773](https://www.ncbi.nlm.nih.gov/genome/1758?genome_assembly_id=648391) | [CABIHU01](https://www.ncbi.nlm.nih.gov/Traces/wgs/?val=CABIHU01) | GCA_902173115.1 | *Y. rohdei* |
|  | *Y. rohdei* | [ATCC 43380](https://www.ncbi.nlm.nih.gov/genome/1758?genome_assembly_id=171623) | [ACCD01](https://www.ncbi.nlm.nih.gov/Traces/wgs/?val=ACCD01) | GCA_000173775.1 | *Y. rohdei* |
|  | *Y. rohdei* | [SCPM-O-B-7599](https://www.ncbi.nlm.nih.gov/genome/1758?genome_assembly_id=321584) | [MWTN01](https://www.ncbi.nlm.nih.gov/Traces/wgs/?val=MWTN01) | GCA_002192875.1 | *Y. rohdei* |
|  | *Y. kristensenii* | [NCTC11471](https://www.ncbi.nlm.nih.gov/genome/875?genome_assembly_id=392058) | [UHIY01](https://www.ncbi.nlm.nih.gov/Traces/wgs/?val=UHIY01) | GCA_900460525.1 | *Y. kristensenii* |
|  | *Y. kristensenii* | [2012N-4030](https://www.ncbi.nlm.nih.gov/genome/875?genome_assembly_id=906993) | [CP054049](https://www.ncbi.nlm.nih.gov/nuccore/CP054049.1) | GCA_013282785.1 | *Y. kristensenii* |
|  | *Y. kristensenii* | [ATCC 33639](https://www.ncbi.nlm.nih.gov/genome/875?genome_assembly_id=207193) | [CP008955](https://www.ncbi.nlm.nih.gov/nuccore/CP008955.1) | GCA_000750355.1 | *Y. rochesterensis* |
|  | *Y. kristensenii* | [FDAARGOS_415](https://www.ncbi.nlm.nih.gov/genome/875?genome_assembly_id=342901) | [PDEI01](https://www.ncbi.nlm.nih.gov/Traces/wgs/?val=PDEI01) | GCA_002554365.1 | *Y. kristensenii* |
|  | *Y. kristensenii* | [FE80982](https://www.ncbi.nlm.nih.gov/genome/875?genome_assembly_id=239511) | [CQAQ01](https://www.ncbi.nlm.nih.gov/Traces/wgs/?val=CQAQ01) | GCA_001158505.1 | *Y. rochesterensis* |
|  | *Y. kristensenii* | [MGYG-HGUT-02462](https://www.ncbi.nlm.nih.gov/genome/875?genome_assembly_id=656843) | [CABMMB01](https://www.ncbi.nlm.nih.gov/Traces/wgs/?val=CABMMB01) | GCA_902387475.1 | *Y. rochesterensis* |
|  | *Y. kristensenii* | [IP38952](https://www.ncbi.nlm.nih.gov/genome/875?genome_assembly_id=647919) | [CABHXR01](https://www.ncbi.nlm.nih.gov/Traces/wgs/?val=CABHXR01) | GCA_902170425.1 | *Y. kristensenii* |
|  | *Y. kristensenii* | [M73](https://www.ncbi.nlm.nih.gov/genome/875?genome_assembly_id=1666857) | [JACCJK01](https://www.ncbi.nlm.nih.gov/Traces/wgs/?val=JACCJK01) | GCA_019425795.1 | *Y. kristensenii* |
|  | *Y. kristensenii* | [M47](https://www.ncbi.nlm.nih.gov/genome/875?genome_assembly_id=1666858) | [JACCJL01](https://www.ncbi.nlm.nih.gov/Traces/wgs/?val=JACCJL01) | GCA_019425855.1 | *Y. kristensenii* |
|  | *Y. kristensenii* | [119/84](https://www.ncbi.nlm.nih.gov/genome/875?genome_assembly_id=239510) | [CPXZ01](https://www.ncbi.nlm.nih.gov/Traces/wgs/?val=CPXZ01) | GCA_001153365.1 | *Y. kristensenii* |
|  | *Y. kristensenii* | [MGYG-HGUT-02461](https://www.ncbi.nlm.nih.gov/genome/875?genome_assembly_id=656844) | [CABMLW01](https://www.ncbi.nlm.nih.gov/Traces/wgs/?val=CABMLW01) | GCA_902387365.1 | *Y. kristensenii* |
|  | *Y. kristensenii* | [OK6311](https://www.ncbi.nlm.nih.gov/genome/875?genome_assembly_id=244211) | [CWJK01](https://www.ncbi.nlm.nih.gov/Traces/wgs/?val=CWJK01) | GCA_001244605.1 | *Y. rochesterensis* |
|  | *Y. kristensenii* | [FCF221](https://www.ncbi.nlm.nih.gov/genome/875?genome_assembly_id=239505) | [CQAG01](https://www.ncbi.nlm.nih.gov/Traces/wgs/?val=CQAG01) | GCA_001091065.1 | *Y. kristensenii* |
|  | *Y. kristensenii* | [CFSAN060538](https://www.ncbi.nlm.nih.gov/genome/875?genome_assembly_id=321080) | [NHOG01](https://www.ncbi.nlm.nih.gov/Traces/wgs/?val=NHOG01) | GCA_002188915.1 | *Y. kristensenii* |
|  | *Y. kristensenii* | [IP39338](https://www.ncbi.nlm.nih.gov/genome/875?genome_assembly_id=647922) | [CABHXL01](https://www.ncbi.nlm.nih.gov/Traces/wgs/?val=CABHXL01) | GCA_902170495.1 | *Y. kristensenii* |
|  | *Y. kristensenii* | [HR100](https://www.ncbi.nlm.nih.gov/genome/875?genome_assembly_id=1666859) | [JACCJW01](https://www.ncbi.nlm.nih.gov/Traces/wgs/?val=JACCJW01) | GCA_019426035.1 | *Y. kristensenii* |
|  | *Y. kristensenii* | [M75](https://www.ncbi.nlm.nih.gov/genome/875?genome_assembly_id=1666860) | [JACCJJ01](https://www.ncbi.nlm.nih.gov/Traces/wgs/?val=JACCJJ01) | GCA_019425815.1 | *Y. kristensenii* |
|  | *Y. kristensenii* | [M70](https://www.ncbi.nlm.nih.gov/genome/875?genome_assembly_id=1666861) | [JACCJU01](https://www.ncbi.nlm.nih.gov/Traces/wgs/?val=JACCJU01) | GCA_019426045.1 | *Y. kristensenii* |
|  | *Y. kristensenii* | [FCF580](https://www.ncbi.nlm.nih.gov/genome/875?genome_assembly_id=239509) | [CQEO01](https://www.ncbi.nlm.nih.gov/Traces/wgs/?val=CQEO01) | GCA_001144805.1 | *Y. kristensenii* |
|  | *Y. kristensenii* | [FCF326](https://www.ncbi.nlm.nih.gov/genome/875?genome_assembly_id=239508) | [CPYI01](https://www.ncbi.nlm.nih.gov/Traces/wgs/?val=CPYI01) | GCA_001134585.1 | *Y. kristensenii* |
|  | *Y. kristensenii* | [IP36506](https://www.ncbi.nlm.nih.gov/genome/875?genome_assembly_id=647918) | [CABHXV01](https://www.ncbi.nlm.nih.gov/Traces/wgs/?val=CABHXV01) | GCA_902170415.1 | *Y. kristensenii* |
|  | *Y. kristensenii* | [IP24139](https://www.ncbi.nlm.nih.gov/genome/875?genome_assembly_id=253697) | [CGCG01](https://www.ncbi.nlm.nih.gov/Traces/wgs/?val=CGCG01) | GCA_001319625.1 | *Y. kristensenii* |
|  | *Y. kristensenii* | [CFSAN060539](https://www.ncbi.nlm.nih.gov/genome/875?genome_assembly_id=911795) | [NHOF01](https://www.ncbi.nlm.nih.gov/Traces/wgs/?val=NHOF01) | GCA_002188895.1 | *Y. hibernica* |
|  | *Y. kristensenii* | [FCF324](https://www.ncbi.nlm.nih.gov/genome/875?genome_assembly_id=239504) | [CQDL01](https://www.ncbi.nlm.nih.gov/Traces/wgs/?val=CQDL01) | GCA_001085865.1 | *Y. kristensenii* |
|  | *Y. kristensenii* | [IP28590](https://www.ncbi.nlm.nih.gov/genome/875?genome_assembly_id=647926) | [CABHXN01](https://www.ncbi.nlm.nih.gov/Traces/wgs/?val=CABHXN01) | GCA_902170575.1 | *Y. kristensenii* |
|  | *Y. kristensenii* | [IP28581](https://www.ncbi.nlm.nih.gov/genome/875?genome_assembly_id=647924) | [CABHXY01](https://www.ncbi.nlm.nih.gov/Traces/wgs/?val=CABHXY01) | GCA_902170535.1 | *Y. rochesterensis* |
|  | *Y. kristensenii* | [ATCC 33638](https://www.ncbi.nlm.nih.gov/genome/875?genome_assembly_id=169813) | [ACCA01](https://www.ncbi.nlm.nih.gov/Traces/wgs/?val=ACCA01) | GCA_000173715.1 | *Y. kristensenii* |
|  | *Y. kristensenii* | [SCPM-O-B-7962 (C-136)](https://www.ncbi.nlm.nih.gov/genome/875?genome_assembly_id=839213) | [JAASAL01](https://www.ncbi.nlm.nih.gov/Traces/wgs/?val=JAASAL01) | GCA_011765215.1 | *Y. kristensenii* |
|  | *Y. kristensenii* | [SCPM-O-B-7961 (C-135)](https://www.ncbi.nlm.nih.gov/genome/875?genome_assembly_id=839214) | [JAASAM01](https://www.ncbi.nlm.nih.gov/Traces/wgs/?val=JAASAM01) | GCA_011765235.1 | *Y. kristensenii* |
|  | *Y. kristensenii* | [SCPM-O-B-7953](https://www.ncbi.nlm.nih.gov/genome/875?genome_assembly_id=354260) | [PGLT01](https://www.ncbi.nlm.nih.gov/Traces/wgs/?val=PGLT01) | GCA_002794215.1 | *Y. kristensenii* |
|  | *Y. kristensenii* | [SCPM-O-B-3969](https://www.ncbi.nlm.nih.gov/genome/875?genome_assembly_id=354736) | [PGWV01](https://www.ncbi.nlm.nih.gov/Traces/wgs/?val=PGWV01) | GCA_002795325.1 | *Y. kristensenii* |
|  | *Y. kristensenii* | [SCPM-O-B-8071](https://www.ncbi.nlm.nih.gov/genome/875?genome_assembly_id=351702) | [PEHL01](https://www.ncbi.nlm.nih.gov/Traces/wgs/?val=PEHL01) | GCA_002738465.1 | *Y. kristensenii* |
|  | *Y. kristensenii* | [SCPM-O-B-7606](https://www.ncbi.nlm.nih.gov/genome/875?genome_assembly_id=321579) | [MWTL01](https://www.ncbi.nlm.nih.gov/Traces/wgs/?val=MWTL01) | GCA_002192915.1 | *Y. kristensenii* |
|  | *Y. kristensenii* | SCPM-O-B-5043 (672) | JAQIRW01 | GCF_027946975.1 | *Y. kristensenii* |
|  | *Y. kristensenii* | SCPM-O-B-8023 (C-189) | JAQIRV01 | GCA_027946955.1 | *Y. kristensenii* |
|  | *Y. kristensenii* | SCPM-O-B-8021 (C-140) | JAQIRU01 | GCA_027946995.1 | *Y. kristensenii* |
|  | *Y. kristensenii* | SCPM-O-B-8023 (C-142) | JAQIRT01 | GCA_027946915.1 | *Y. kristensenii* |
|  | *Y. kristensenii* | SCPM-O-B-8022 (C-141) | JAQISE01 | GCA_027947125.1 | *Y. kristensenii* |
|  | *Y. massiliensis* | [GTA](https://www.ncbi.nlm.nih.gov/genome/14388?genome_assembly_id=371871) | CP028487 | GCA_003048255.1 | *Y. massiliensis* |
|  | *Y. massiliensis* | [2011N-4075](https://www.ncbi.nlm.nih.gov/genome/14388?genome_assembly_id=907005) | CP054048 | GCA_013282765.1 | *Y. massiliensis* |
|  | *Y. massiliensis* | [CIP109351](https://www.ncbi.nlm.nih.gov/genome/14388?genome_assembly_id=648654) | [CABHYH01](https://www.ncbi.nlm.nih.gov/Traces/wgs/?val=CABHYH01) | GCA_902170765.1 | *Y. massiliensis* |
|  | *Y. massiliensis* | [IZSPB_Y116](https://www.ncbi.nlm.nih.gov/genome/14388?genome_assembly_id=1723621) | [JAJAWB01](https://www.ncbi.nlm.nih.gov/Traces/wgs/?val=JAJAWB01) | GCA_020531745.1 | *Y. massiliensis* |
|  | *Y. massiliensis* | [IZSPB_Y100](https://www.ncbi.nlm.nih.gov/genome/14388?genome_assembly_id=1723622) | [JAJAWA01](https://www.ncbi.nlm.nih.gov/Traces/wgs/?val=JAJAWA01) | GCA_020531815.1 | *Y. massiliensis* |
|  | *Y. massiliensis* | [IP39254](https://www.ncbi.nlm.nih.gov/genome/14388?genome_assembly_id=648651) | [CABHYC01](https://www.ncbi.nlm.nih.gov/Traces/wgs/?val=CABHYC01) | GCA_902170685.1 | *Y. massiliensis* |
|  | *Y. massiliensis* | [IP37093](https://www.ncbi.nlm.nih.gov/genome/14388?genome_assembly_id=648649) | [CABHYT01](https://www.ncbi.nlm.nih.gov/Traces/wgs/?val=CABHYT01) | GCA_902170615.1 | *Y. massiliensis* |
|  | *Y. massiliensis* | [IP39269](https://www.ncbi.nlm.nih.gov/genome/14388?genome_assembly_id=648650) | [CABHYR01](https://www.ncbi.nlm.nih.gov/Traces/wgs/?val=CABHYR01) | GCA_902170655.1 | *Y. massiliensis* |
|  | *Y. massiliensis* | [CIP109352](https://www.ncbi.nlm.nih.gov/genome/14388?genome_assembly_id=648652) | [CABHYQ01](https://www.ncbi.nlm.nih.gov/Traces/wgs/?val=CABHYQ01) | GCA_902170715.1 | *Y. massiliensis* |
|  | *Y. massiliensis* | [24070](https://www.ncbi.nlm.nih.gov/genome/14388?genome_assembly_id=239585) | [CQBH01](https://www.ncbi.nlm.nih.gov/Traces/wgs/?val=CQBH01) | GCA_001142305.1 | *Y. massiliensis* |
|  | *Y. massiliensis* | [IP34847](https://www.ncbi.nlm.nih.gov/genome/14388?genome_assembly_id=648653) | [CABHYD01](https://www.ncbi.nlm.nih.gov/Traces/wgs/?val=CABHYD01) | GCA_902170735.1 | *Y. massiliensis* |
|  | *Y. massiliensis* | [CCUG 53443](https://www.ncbi.nlm.nih.gov/genome/14388?genome_assembly_id=47576) | [CAKR01](https://www.ncbi.nlm.nih.gov/Traces/wgs/?val=CAKR01) | GCA_000312485.1 | *Y. massiliensis* |
|  | *Y. massiliensis* | [SCPM-O-B-8024](https://www.ncbi.nlm.nih.gov/genome/14388?genome_assembly_id=351847) | [PEHM01](https://www.ncbi.nlm.nih.gov/Traces/wgs/?val=PEHM01) | GCA_002738425.1 | *Y. massiliensis* |
|  | *Y. massiliensis* | [SCPM-O-B-802](https://www.ncbi.nlm.nih.gov/genome/14388?genome_assembly_id=351847)5 | JAASAN01 | GCA_011765335.1 | *Y. massiliensis* |
|  | *Y. massilensis* | SCPM-O-B-8026 (C-146) | JAQISG01 | GCA_027947195.1 | *Y. massilensis* |
|  | *Y. aldovae* | IP07971 | CABHQA01 | GCA_902168585.1 | *Y. aldovae* |
|  | *Y. aldovae* | IP08631 | CABHQG01 | GCA_902168525.1 | *Y. aldovae* |
|  | *Y. aldovae* | IP08789 | CABHPY01 | GCA_902168495.1 | *Y. aldovae* |
|  | *Y. aldovae* | IP08290 | CABHQC01 | GCA_902168475.1 | *Y. aldovae* |
|  | *Y. aldovae* | IP06005 | CQEJ01 | GCA_001091225.1 | *Y. aldovae* |
|  | *Y. aldovae* | IP07632 | CQAX01 | GCA_001122605.1 | *Y. aldovae* |
|  | *Y. aldovae* | IP08791 | CQEH01 | GCA_001139945.1 | *Y. aldovae* |
|  | *Y. aldovae* | IP23238 | CQCP01 | GCA_001164505.1 | *Y. aldovae* |
|  | *Y. aldovae* | IP08619 | CABHQE01 | GCA_902168455.1 | *Y. aldovae* |
|  | *Y. aldovae* | 670-83 | CP009781 | GCA_000834395.1 | *Y. aldovae* |
|  | *Y. aldovae* | ATCC 35236 | ACCB01 | GCA_000173735.1 | *Y. aldovae* |
|  | *Y. entomophaga* | [MH96](https://www.ncbi.nlm.nih.gov/genome/45128?genome_assembly_id=276925) | CP010029 | GCA_001656035.1 | *Y. entomophaga* |
|  | *Y. entomophaga* | [SCPM-O-B-7183](https://www.ncbi.nlm.nih.gov/genome/45128?genome_assembly_id=321601) | [MWTM01](https://www.ncbi.nlm.nih.gov/Traces/wgs/?val=MWTM01) | GCA_002192845.1 | *Y. entomophaga* |
|  | *Y. vastinensis* | IP37831 | CABHWP01 | GCA_902170405.1 | *Y. vastinensis* |
|  | *Y. vastinensis* | IP38594 | CACVAF01 | GCA_902726565.1 | *Y. vastinensis* |
|  | *Y. vastinensis* | IP38178 | CABHWT01 | GCA_902170255.1 | *Y. vastinensis* |
|  | *Y. vastinensis* | IP38006 | CABHXG01 | GCA_902170245.1 | *Y. vastinensis* |
|  | *Y. vastinensis* | IP38831 | CABHXD01 | GCA_902170295.1 | *Y. vastinensis* |
|  | *Y. alsatica* | IP38850 | CABHWW01 | GCA_902170305.1 | *Y. alsatica* |
|  | *Y. alsatica* | IP38166 | CABHWQ01 | GCA_902170345.1 | *Y. alsatica* |
|  | *Y. alsatica* | IP37802 | CABHWS01 | GCA_902170375.1 | *Y. alsatica* |
|  | *Y. alsatica* | IP39458 | CABHWY01 | GCA_902170285.1 | *Y. alsatica* |
|  | *Y. alsatica* | IP37124 | CABHWO01 | GCA_902170395.1 | *Y. alsatica* |
|  | *Y. alsatica* | IP39797 | CABHXE01 | GCA_902170325.1 | *Y. alsatica* |
|  | *Y. alsatica* | IP38403 | CABHXC01 | GCA_902170385.1 | *Y. alsatica* |
|  | *Y. alsatica* | IP38767 | CABHWX01 | GCA_902170275.1 | *Y. alsatica* |
|  | *Y. alsatica* | IP35553 | CABHWR01 | GCA_902170365.1 | *Y. alsatica* |
|  | *Y. alsatica* | SCPM-O-B-7604 | CP104006 | GCA_025133195.1 | *Y. alsatica* |
|  | *Y. proxima* | IP38046 | CABHZF01 | GCA_902171005.1 | *Y. proxima* |
|  | *Y. proxima* | IP38819 | CABHZI01 | GCA_902170975.1 | *Y. proxima* |
|  | *Y. proxima* | IP39432 | CABHZN01 | GCA_902170955.1 | *Y. proxima* |
|  | *Y. proxima* | IP37838 | CABHYU01 | GCA_902170665.1 | *Y. proxima* |
|  | *Y. proxima* | IP38868 | CABHZB01 | GCA_902170815.1 | *Y. proxima* |
|  | *Y. proxima* | IP38191 | CABHYX01 | GCA_902170985.1 | *Y. proxima* |
|  | *Y. proxima* | IP38663 | CABHZK01 | GCA_902170895.1 | *Y. proxima* |
|  | *Y. proxima* | IP39924 | CABHZG01 | GCA_902170885.1 | *Y. proxima* |
|  | *Y. proxima* | IP38950 | CABHYW01 | GCA_902170945.1 | *Y. proxima* |
|  | *Y. proxima* | IP37424 | CABHYG01 | GCA_902170785.1 | *Y. proxima* |
|  | *Y. artesiana* | IP41384 | CACVAC01 | GCA_902726525.1 | *Y. artesiana* |
|  | *Y. artesiana* | IP42281 | CACVAD01 | GCA_902726545.1 | *Y. artesiana* |
|  | *Y. artesiana* | IP42750 | CACVAE01 | GCA_902726535.1 | *Y. artesiana* |
|  | *Y. artesiana* | IP39904 | CABHYL01 | GCA_902170805.1 | *Y. artesiana* |
|  | *Y. thracica* | IP35448 | CABHXQ01 | GCA_902170455.1 | *Y. thracica* |
|  | *Y. thracica* | IP42199 | CACVAB01 | GCA_902726555.1 | *Y. thracica* |
|  | *Y. thracica* | IP6945 | CQAW01 | GCA_001123825.1 | *Y. thracica* |
|  | *Y. thracica* | IP34646 | CABHXX01 | GCA_902170565.1 | *Y. thracica* |
|  | *Y. rochesterensis* | IP38487 | CABHXM01 | GCA_902170595.1 | *Y. rochesterensis* |
|  | *Y. rochesterensis* | IP38810 | CABHXZ01 | GCA_902170465.1 | *Y. rochesterensis* |
|  | *Y. rochesterensis* | IP37484 | CABHXK01 | GCA_902170505.1 | *Y. rochesterensis* |
|  | *Y. rochesterensis* | IP38921 | CABHXT01 | GCA_902170475.1 | *Y. rochesterensis* |
|  | *Y. rochesterensis* | ATCC BAA-2637 | CP032482 | GCA_003600645.1 | *Y. rochesterensis* |
|  | *Y. rochesterensis* | IP35638 | CABHXS01 | GCA_902170605.1 | *Y. rochesterensis* |
|  | *Y. rochesterensis* | [Y231](https://www.ncbi.nlm.nih.gov/genome/875?genome_assembly_id=219472) | [CP009997](https://www.ncbi.nlm.nih.gov/nuccore/CP009997.1) | GCA_000834865.1 | *Y. rochesterensis* |
|  | *Y. rochesterensis* | SCPM-O-B-9106 (C-191) | JAQISF01 | GCA_027947175.1 | *Y. rochesterensis* |
|  | *Y. canariae* | NCTC 14382 | CP043727 | GCA_009831415.1 | *Y. canariae* |
|  | *Y. canariae* | IP38017 | CABHYP01 | GCA_902170705.1 | *Y. canariae* |
|  | *Y. canariae* | IP37834 | CABHYN01 | GCA_902170775.1 | *Y. canariae* |
|  | *Y. hibernica* | CFS1934 | CP032487 | GCA_004124235.1 | *Y. hibernica* |
|  | *Y. hibernica* | IP37048 | CABHXI01 | GCA_902170435.1 | *Y. hibernica* |
|  | *Y. nurmii* | CIP110231T | CPYD01 | GCA_001112925.1 | *Y. nurmii* |
|  | *Y. pekkanenii* | CIP110230 | CWJL01 | GCA_001244635.1 | *Y. pekkanenii* |
|  | *Y. pekkanenii* | A125KOH2 | CQAZ01 | GCA_001152565.1 | *Y. pekkanenii* |
|  | *Y. aleksiciae* | [404/81](https://www.ncbi.nlm.nih.gov/genome/38751?genome_assembly_id=254019) | [CGBL01](https://www.ncbi.nlm.nih.gov/Traces/wgs/?val=CGBL01) | GCA_001319845.1 | *Y. aleksiciae* |
|  | *Y. aleksiciae* | [159](https://www.ncbi.nlm.nih.gov/genome/38751?genome_assembly_id=233887) | CP011975 | GCA_001047675.1 | *Y. aleksiciae* |
|  | *Y. aleksiciae* | [IP27925](https://www.ncbi.nlm.nih.gov/genome/38751?genome_assembly_id=964876) | [CQEM01](https://www.ncbi.nlm.nih.gov/Traces/wgs/?val=CQEM01) | GCA_001115185.1 | *Y. aleksiciae* |
|  | *Y. aleksiciae* | [MGYG-HGUT-02458](https://www.ncbi.nlm.nih.gov/genome/38751?genome_assembly_id=657742) | [CABMLM01](https://www.ncbi.nlm.nih.gov/Traces/wgs/?val=CABMLM01) | GCA_902387205.1 | *Y. aleksiciae* |
|  | *Y. aleksiciae* | [IP38862](https://www.ncbi.nlm.nih.gov/genome/38751?genome_assembly_id=648761) | [CABHPU01](https://www.ncbi.nlm.nih.gov/Traces/wgs/?val=CABHPU01) | GCA_902168515.1 | *Y. aleksiciae* |
|  | *Y. aleksiciae* | [IP29443](https://www.ncbi.nlm.nih.gov/genome/38751?genome_assembly_id=648760) | [CABHPX01](https://www.ncbi.nlm.nih.gov/Traces/wgs/?val=CABHPX01) | GCA_902168465.1 | *Y. aleksiciae* |
|  | *Y. aleksiciae* | [IP28587](https://www.ncbi.nlm.nih.gov/genome/38751?genome_assembly_id=648762) | [CABHQK01](https://www.ncbi.nlm.nih.gov/Traces/wgs/?val=CABHQK01) | GCA_902168595.1 | *Y. aleksiciae* |
|  | *Y. aleksiciae* | [IP28585](https://www.ncbi.nlm.nih.gov/genome/38751?genome_assembly_id=648763) | [CABHQI01](https://www.ncbi.nlm.nih.gov/Traces/wgs/?val=CABHQI01) | GCA_902168605.1 | *Y. aleksiciae* |
|  | *Y. aleksiciae* | [IP28584](https://www.ncbi.nlm.nih.gov/genome/38751?genome_assembly_id=648759) | [CABHQD01](https://www.ncbi.nlm.nih.gov/Traces/wgs/?val=CABHQD01) | GCA_902168445.1 | *Y. aleksiciae* |
|  | *Y. aleksiciae* | [SCPM-O-B-8086 (J332)](https://www.ncbi.nlm.nih.gov/genome/38751?genome_assembly_id=839223) | [JAASAK01](https://www.ncbi.nlm.nih.gov/Traces/wgs/?val=JAASAK01) | GCA_011765225.1 | *Y. aleksiciae* |
|  | *Y. aleksiciae* | SCPM-O-B-8087 (216) | JAQISD01 | GCA_027947155.1 | *Y. aleksiciae* |
|  | *Y. similis* | 228 | CP007230 | GCA_000582515.1 | *Y. similis* |
|  | *Y. similis* | MW332-1 | CABIIH01 | GCA_902173095.1 | *Y. similis* |
|  | *Y. similis* | Y228 | CGBP01 | GCA_001053095.1 | *Y. similis* |
|  | *Y. similis* | R819 | CPZI01 | GCA_001140625.1 | *Y. similis* |
|  | *Y. similis* | Y233 | CQBK01 | GCA_001114105.1 | *Y. similis* |
|  | *Y. similis* | MW109-2 | CPYL01 | GCA_001136465.1 | *Y. similis* |
|  | *Y. similis* | Y252 | CHJS01 | GCA_001319745.1 | *Y. similis* |
|  | *Y. similis* | OK6609 | CABIHS01 | GCA_902173255.1 | *Y. similis* |
|  | *Y. similis* | Kuratani-2 | CABIHV01 | GCA_902173105.1 | *Y. similis* |
|  | *Y. ruckeri* | 17Y0159 | CP084643 | GCA_021399135.1 | *Y. ruckeri* |
|  | *Y. ruckeri* | 17Y0414 | CP084635 | GCA_021399075.1 | *Y. ruckeri* |
|  | *Y. ruckeri* | 17Y0163 | CP084641 | GCA_021399115.1 | *Y. ruckeri* |
|  | *Y. ruckeri* | NHV_3758 | CP023184 | GCA_002442495.2 | *Y. ruckeri* |
|  | *Y. ruckeri* | 17Y0157 | CP084647 | GCA_021399195.1 | *Y. ruckeri* |
|  | *Y. ruckeri* | Big Creek 74 | CP011078 | GCA_000964565.1 | *Y. ruckeri* |
|  | *Y. ruckeri* | 16Y0180 | CP084652 | GCA_021399215.1 | *Y. ruckeri* |
|  | *Y. ruckeri* | 17Y0161 | CP084642 | GCA_021399155.1 | *Y. ruckeri* |
|  | *Y. ruckeri* | YRB | CP009539 | GCA_000834255.1 | *Y. ruckeri* |
|  | *Y. ruckeri* | QMA0440 | CP017236 | GCA_002192595.1 | *Y. ruckeri* |
|  | *Y. ruckeri* | 17Y0412 | CP084637 | GCA_021399055.1 | *Y. ruckeri* |
|  | *Y. ruckeri* | 17Y0189 | CP084639 | GCA_021399095.1 | *Y. ruckeri* |
|  | *Y. ruckeri* | SC09 | CP025800 | GCA_000775355.2 | *Y. ruckeri* |
|  | *Y. ruckeri* | 17Y0153 | CP084650 | GCA_021399175.1 | *Y. ruckeri* |
|  | *Y. ruckeri* | 17Y0155 | CP084648 | GCA_021399235.1 | *Y. ruckeri* |
|  | *Y. ruckeri* | KMM821 | CP071802 | GCA_017498685.1 | *Y. ruckeri* |
|  | *Y. ruckeri* | 93/1038-1 | MDZU01 | GCA_001882715.1 | *Y. ruckeri* |
|  | *Y. ruckeri* | 07/3828-6E | MECF01 | GCA_001883105.1 | *Y. ruckeri* |
|  | *Y. ruckeri* | 01/0298 | MEAG01 | GCA_001883425.1 | *Y. ruckeri* |
|  | *Y. ruckeri* | 04/2640-8k | MEAO01 | GCA_001883605.1 | *Y. ruckeri* |
|  | *Y. ruckeri* | Feb-00 | MEAK01 | GCA_001883535.1 | *Y. ruckeri* |
|  | *Y. ruckeri* | AHL6 | MEBT01 | GCA_001883025.1 | *Y. ruckeri* |
|  | *Y. ruckeri* | AHL1 | MEBY01 | GCA_001883185.1 | *Y. ruckeri* |
|  | *Y. ruckeri* | AHL5 | MEBU01 | GCA_001882995.1 | *Y. ruckeri* |
|  | *Y. ruckeri* | 05/0285-1K | MEAP01 | GCA_001883615.1 | *Y. ruckeri* |
|  | *Y. ruckeri* | 04/1779 | MEAN01 | GCA_001883575.1 | *Y. ruckeri* |
|  | *Y. ruckeri* | 00/2994 | MEAE01 | GCA_001883405.1 | *Y. ruckeri* |
|  | *Y. ruckeri* | 14/0125-1k | MEBZ01 | GCA_001883155.1 | *Y. ruckeri* |
|  | *Y. ruckeri* | 00/0652-K3 | MEAB01 | GCA_001883275.1 | *Y. ruckeri* |
|  | *Y. ruckeri* | Mar-05 | MEAL01 | GCA_001883525.1 | *Y. ruckeri* |
|  | *Y. ruckeri* | 02/0981-4br | MEAI01 | GCA_001883485.1 | *Y. ruckeri* |
|  | *Y. ruckeri* | 09/0217-5k | MECD01 | GCA_001882945.1 | *Y. ruckeri* |
|  | *Y. ruckeri* | IP27754 | CABIHT01 | GCA_902173085.1 | *Y. ruckeri* |
|  | *Y. ruckeri* | 08/0188-3K | MECE01 | GCA_001882925.1 | *Y. ruckeri* |
|  | *Y. ruckeri* | NCTC12266 | JAJIBR01 | GCA_023212225.1 | *Y. ruckeri* |
|  | *Y. ruckeri* | AHL7 | MEBS01 | GCA_001883035.1 | *Y. ruckeri* |
|  | *Y. ruckeri* | OMBL4 | CPUZ01 | GCA_001172905.1 | *Y. ruckeri* |
|  | *Y. ruckeri* | RS41 | CQBN01 | GCA_001166725.1 | *Y. ruckeri* |
|  | *Y. ruckeri* | 150 | MKFJ01 | GCA_001750505.1 | *Y. ruckeri* |
|  | *Y. ruckeri* | NVI-10990 | JAJIBN01 | GCA_023212395.1 | *Y. ruckeri* |
|  | *Y. ruckeri* | 97/1152-1 | MDZZ01 | GCA_001883265.1 | *Y. ruckeri* |
|  | *Y. ruckeri* | NVI-3629 | JAJIBH01 | GCA_023212465.1 | *Y. ruckeri* |
|  | *Y. ruckeri* | 87/3421-SP | MDZJ01 | GCA_001880465.1 | *Y. ruckeri* |
|  | *Y. ruckeri* | NVI-10587 | JAJIBL01 | GCA_023212425.1 | *Y. ruckeri* |
|  | *Y. ruckeri* | NVI-10974 | JAJIBM01 | GCA_023212365.1 | *Y. ruckeri* |
|  | *Y. ruckeri* | NVI-494 | JAJIBF01 | GCA_023212525.1 | *Y. ruckeri* |
|  | *Y. ruckeri* | NVI-9967 | JAJIBK01 | GCA_023212475.1 | *Y. ruckeri* |
|  | *Y. ruckeri* | 00/1793-k2 | MEAD01 | GCA_001883365.1 | *Y. ruckeri* |
|  | *Y. ruckeri* | 11/4666-4k | MECB01 | GCA_001883115.1 | *Y. ruckeri* |
|  | *Y. ruckeri* | 07/3342-1k | MECH01 | GCA_001882895.1 | *Y. ruckeri* |
|  | *Y. ruckeri* | 02/0972-2br | MEAH01 | GCA_001883435.1 | *Y. ruckeri* |
|  | *Y. ruckeri* | AHL3 | MEBW01 | GCA_001882975.1 | *Y. ruckeri* |
|  | *Y. ruckeri* | 12/3871-3K | MECA01 | GCA_001883145.1 | *Y. ruckeri* |
|  | *Y. ruckeri* | NCTC12268 | JAJIBS01 | GCA_023212305.1 | *Y. ruckeri* |
|  | *Y. ruckeri* | NVI-11050 | JAJIBP01 | GCA_023212385.1 | *Y. ruckeri* |
|  | *Y. ruckeri* | NCTC12270 | JAJIBU01 | GCA_023212235.1 | *Y. ruckeri* |
|  | *Y. ruckeri* | 88/3837 | MDZK01 | GCA_001880365.1 | *Y. ruckeri* |
|  | *Y. ruckeri* | 88/4281-4 | MDZM01 | GCA_001880425.1 | *Y. ruckeri* |
|  | *Y. ruckeri* | 93/5839-1 | MDZV01 | GCA_001882465.1 | *Y. ruckeri* |
|  | *Y. ruckeri* | 95/6654-1 | MDZX01 | GCA_001883245.1 | *Y. ruckeri* |
|  | *Y. ruckeri* | 95/4881-1 | MDZW01 | GCA_001883335.1 | *Y. ruckeri* |
|  | *Y. ruckeri* | 89/3717-10 | MDZN01 | GCA_001880475.1 | *Y. ruckeri* |
|  | *Y. ruckeri* | 37551 | JPFO01 | GCA_000737165.1 | *Y. ruckeri* |
|  | *Y. ruckeri* | 05/0297-5eye | MECI01 | GCA_001883065.1 | *Y. ruckeri* |
|  | *Y. ruckeri* | 07/3642-5K-b | MECG01 | GCA_001883075.1 | *Y. ruckeri* |
|  | *Y. ruckeri* | 97/0226-1 vc | MEAA01 | GCA_001883345.1 | *Y. ruckeri* |
|  | *Y. ruckeri* | 01/0230-7 | MEAF01 | GCA_001883415.1 | *Y. ruckeri* |
|  | *Y. ruckeri* | Feb-68 | MEAJ01 | GCA_001883495.1 | *Y. ruckeri* |
|  | *Y. ruckeri* | 04/1749 | MEAM01 | GCA_001883565.1 | *Y. ruckeri* |
|  | *Y. ruckeri* | 89/4243 | MDZO01 | GCA_001880435.1 | *Y. ruckeri* |
|  | *Y. ruckeri* | 92/5354-1 | MDZT01 | GCA_001882625.1 | *Y. ruckeri* |
|  | *Y. ruckeri* | 91/4316 | MDZS01 | GCA_001882675.1 | *Y. ruckeri* |
|  | *Y. ruckeri* | 96/5134-k | MDZY01 | GCA_001883255.1 | *Y. ruckeri* |
|  | *Y. ruckeri* | 90/4316 | MDZQ01 | GCA_001883745.1 | *Y. ruckeri* |
|  | *Y. ruckeri* | 91/4311 A1 | MDZR01 | GCA_001883755.1 | *Y. ruckeri* |
|  | *Y. ruckeri* | 90/0961-C9 | MDZP01 | GCA_001883765.1 | *Y. ruckeri* |
|  | *Y. ruckeri* | 88/3873 | MDZL01 | GCA_001880355.1 | *Y. ruckeri* |
|  | *Y. ruckeri* | NVI-9681 | JAJIBJ01 | GCA_023212445.1 | *Y. ruckeri* |
|  | *Y. ruckeri* | NCTC12269 | JAJIBT01 | GCA_023212255.1 | *Y. ruckeri* |
|  | *Y. ruckeri* | NVI-11000 | JAJIBO01 | GCA_023212345.1 | *Y. ruckeri* |
|  | *Y. ruckeri* | CSF007-82 | CCYO01 | GCA_000824965.1 | *Y. ruckeri* |
|  | *Y. ruckeri* | NVI-6225 | JAJIBI01 | GCA_023212505.1 | *Y. ruckeri* |
|  | *Y. ruckeri* | AHL2 | MEBX01 | GCA_001883195.1 | *Y. ruckeri* |
|  | *Y. ruckeri* | NVI-1347 | JAJIBG01 | GCA_023212545.1 | *Y. ruckeri* |
|  | *Y. ruckeri* | FMV-22 | VDHI01 | GCA_008086925.1 | *Y. ruckeri* |
|  | *Y. ruckeri* | SCPM-O-B-8085 | PEHK01 | GCA_002738395.1 | *Y. ruckeri* |
|  | *Y. ruckeri* | NCTC12986 | UHJF01 | GCA_900460715.1 | *Y. ruckeri* |
|  | *Y. ruckeri* | NCTC10476 | UHJG01 | GCA_900460675.1 | *Y. ruckeri* |
|  | *Y. ruckeri* | NVI-344 | JAJIBV01 | GCA_023212245.1 | *Y. ruckeri* |
|  | *Y. ruckeri* | NVI-11076 | JAJIBQ01 | GCA_023212325.1 | *Y. ruckeri* |
|  | *Y. ruckeri* | NVI-492 | JAJIBE01 | GCA_023212565.1 | *Y. ruckeri* |
|  | *Y. ruckeri* | AHL4 | MEBV01 | GCA_001883225.1 | *Y. ruckeri* |
|  | *Y. ruckeri* | 11/4175-3k | MECC01 | GCA_001882955.1 | *Y. ruckeri* |
|  | *Y. ruckeri* | IP27752 | CABIHR01 | GCA_902173145.1 | *Y. ruckeri* |
|  | *Y. ruckeri* | NVI-10705 | JAJJIH01 | GCA_023212585.1 | *Y. ruckeri* |
|  | *Y. ruckeri* | 00/1445 | MEAC01 | GCA_001883325.1 | *Y. ruckeri* |
|  | *Y. ruckeri* | ATCC 29473 | JPPT01 | GCA_000754815.1 | *Y. ruckeri* |
|  | *Y. ruckeri* | ATCC 29473 | ACCC01 | GCA_000173755.1 | *Y. ruckeri* |
|  | *Y. ruckeri* | SCPM-O-B-8298 (H529-36/85) | JAQISB01 | GCA_027947075.1 |  |
|  | *Y. wautersii* | WP-931201 | CVMG01 | GCA_001319825.1 | *Y. wautersii* |
|  | *Y. enterocolitica* subsp. *enterocolitica* | 8081 | CP009846 | GCA_000834795.1 | *Y. enterocolitica* |
|  | *Y. enterocolitica* subsp. *palearctica* | Y11 | FR729477 | GCF_000253175.1 | *Y. enterocolitica* |
|  | *Y. pseudotuberculosis* | IP 32953 | CP009712 | GCA_000834295.1 | *Y. pseudotuberculosis* |
|  | *Y. pestis* | CO92 | AL590842 | GCA_000009065.1 | *Y. pestis* |
