# Supplementary material for: Evaluation of 16S rRNA genes sequences and genome-based analysis for identification of non-pathogenic Yersinia
Source: Front Microbiol. 2025 Jan 7;15:1519733. doi: 10.3389/fmicb.2024.1519733 (PMC11753223; doi:10.3389/fmicb.2024.1519733)
Supplement: SUPPLEMENTARY TABLE S2 — Sequencing technologies, assembly methods and NCBI Genome accession numbers of Yersinia complete genomes used in the study. [file Table_2.docx]

Supplementary Table S2 Sequencing technologies, assembly methods and NCBI Genome accession numbers of *Yersinia* complete genomes used in the study

| Strain name | Assembly Method | Sequencing Technology | Assembly  accession no. |
| --- | --- | --- | --- |
| *Y. aldovae* 670-83 | Newbler v. 2.6; Velvet v. 1.2.08; Allpaths v. 44837; Phrap v. SPS - 4.24; HGAP v. 2.2.0 | Illumina; PacBio; 454 | CP009781 |
| *Y. frederiksenii* FDAARGOS_417 | SMRT v. 2.3.0, HGAP v. 3 | PacBio; Illumina | CP023962 |
| *Y. frederiksenii* FDAARGOS_418 | Celera v. 8.2 | PacBio; Illumina | CP023964 |
| *Y. frederiksenii* Y225 | Newbler v. 2.6; Velvet v. 1.2.08; Allpaths v. 44837; Phrap v. SPS - 4.24 | Illumina; 454 | CP009364 |
| *Y. bercovieri* ATCC 43970 | HGAP v. 3 | PacBio RSII | CP054044 |
| *Y. rohdei* YRA | Newbler v. 2.6; Velvet v. 1.2.08; Allpaths v. 44837; Phrap v. SPS - 4.24; HGAP v. 2.2.0 | Illumina; PacBio; 454 | CP009787 |
| *Y. intermedia* FDAARGOS_730 | canu v. 1.4 | Pacbio; Illumina | CP046293 |
| *Y. intermedia* FDAARGOS_729 | SMRT v. 2.3.0, HGAP v. 3 | Pacbio; Illumina | CP046294 |
| *Y. intermedia* N6/293 | HGAP v. 4 | PacBio Sequel | CP093322 |
| *Y. intermedia* Y228 | Newbler v. 2.6; Velvet v. 1.2.08; Allpaths v. 44837; Phrap v. SPS - 4.24; HGAP v. 2.2.0 | Illumina; PacBio; 454 | CP009801 |
| *Y. intermedia* NCTC11469 | SMRT | PacBio RS | LR134116 |
| *Y. intermedia* FDAARGOS_358 | CA v. 8.2 | PacBio; Illumina | CP027397 |
| *Y. mollaretii* ATCC 43969 | HGAP v. 3 | PacBio RSII | CP054043 |
| *Y. rochesterensis* ATCC 33639 | Newbler v. 2.6; Velvet v. 1.2.08; Allpathsv. 39750; Phrap v. SPS - 4.24 | Illumina; 454 | CP008955 |
| *Y. kristensenii* 2012N-4030 | HGAP v. 3 | PacBio RSII | CP054049 |
| *Y. rochesterensis* Y231 | Newbler v. 2.6; Velvet v. 1.2.08; Allpaths v. 44837; Phrap v. SPS - 4.24; HGAP v. 2.2.0 | Illumina; PacBio; 454 | CP009997 |
| *Y. massiliensis* 2011N-4075 | HGAP v. 3 | PacBio RSII | CP054048 |
| *Y. massiliensis* GTA | SPAdes v. 3.11.1; HGAP v. NOV-2016 | PacBio; Illumina MiSeq | CP028487 |
| *Y. entomophaga* MH96 | Velvet v. Nov-2013 | Illumina | CP010029 |
| *Y. alsatica* SCPM-O-B-7604 | Unicycler v. v 0.4.7 | Oxford Nanopore MinION; MGISeq-2000 | CP104006 |
| *Y. similis* 228 | Bioedit v. 06022014 | PacBio | CP007230 |
| *Y. rochesterensis* ATCC BAA-2637 | SPAdes v. JAN-2014 | Illumina MiSeq | CP032482 |
| *Y. aleksiciae* 159 | Geneious v. 8.1.3 | PacBio | CP011975 |
| *Y.* *hibernica* CFS1934 | Unicycler v. 0.4.6 | Illumina HiSeq; Oxford Nanopore MiniION | CP032487 |
| *Y. canariae* NCTC 14382 | Flye v. v2.5 | Oxford Nanopore MiniION; Illumina HiSeq | CP043727 |
| *Y. pestis* CO92 | Artemis | Sanger | AL590842 |
| *Y. pseudotuberculosis* IP 32953 | Newbler v. 2.6; Velvet v. 1.2.08; Allpaths v. 44837; Phrap v. SPS - 4.24; HGAP v. 2.2.0 | Illumina; PacBio; 454 | CP009712 |
| *Y. enterocolitica* 8081 | Newbler v. 2.6; Velvet v. 1.2.08; Allpaths v. 44837; Phrap v. SPS - 4.24; HGAP v. 2.2.0 | Illumina; PacBio; 454 | CP009846 |
| *Y. enterocolitica subsp. palearctica* Y11 |  | PacBio RS | FR729477 |
| *Y. ruckeri* KMM821 | CLC Genomics Workbench v. 20; SPAdes v. 3.13.0 | Illumina MiSeq; Oxford Nanopore MiniION | CP071802 |
| *Y. ruckeri* QMA0440 | not specified | not specified | CP017236 |
| *Y. ruckeri* 17Y0412 | HGAP v. v03 | PacBio RS | CP084637 |
| *Y. ruckeri* 17Y0189 | HGAP v. v03 | PacBio RS | CP084639 |
| *Y. ruckeri* SC09 | Canu v. 1.6 | PacBio | CP025800 |
| *Y. ruckeri* 17Y0153 | HGAP v. v03 | PacBio RS | CP084650 |
| *Y. ruckeri* 17Y0155 | HGAP v. v03 | PacBio RS | CP084648 |
| *Y. ruckeri* 17Y0159 | HGAP v. v03 | PacBio RS | CP084643 |
| *Y. ruckeri* 17Y0414 | HGAP v. v03 | PacBio RS | CP084635 |
| *Y. ruckeri* 17Y0163 | HGAP v. v03 | PacBio RS | CP084641 |
| *Y. ruckeri* NHV_3758 | HGAP v. v3 | PacBio | CP023184 |
| *Y. ruckeri* 17Y0157 | HGAP v. v03 | PacBio RS | CP084647 |
| *Y. ruckeri* Big Creek 74 | Celera assembler & DNASTAR SeqmanPro v. 11 | PacBio; Sanger dideoxy | CP011078 |
| *Y. ruckeri* 16Y0180 | HGAP v. v03 | PacBio RS | CP084652 |
| *Y. ruckeri* 17Y0161 | HGAP v. v03 | PacBio RS | CP084642 |
| *Y. ruckeri* YRB | Newbler v. 2.6; Velvet v. 1.2.08; Allpaths v. 44837; Phrap v. SPS - 4.24 | Illumina; 454 | CP009539 |
