# Supplementary figures and images for: Evaluation of 16S rRNA genes sequences and genome-based analysis for identification of non-pathogenic Yersinia
Source: Front Microbiol. 2025 Jan 7;15:1519733. doi: 10.3389/fmicb.2024.1519733 (PMC11753223; doi:10.3389/fmicb.2024.1519733)

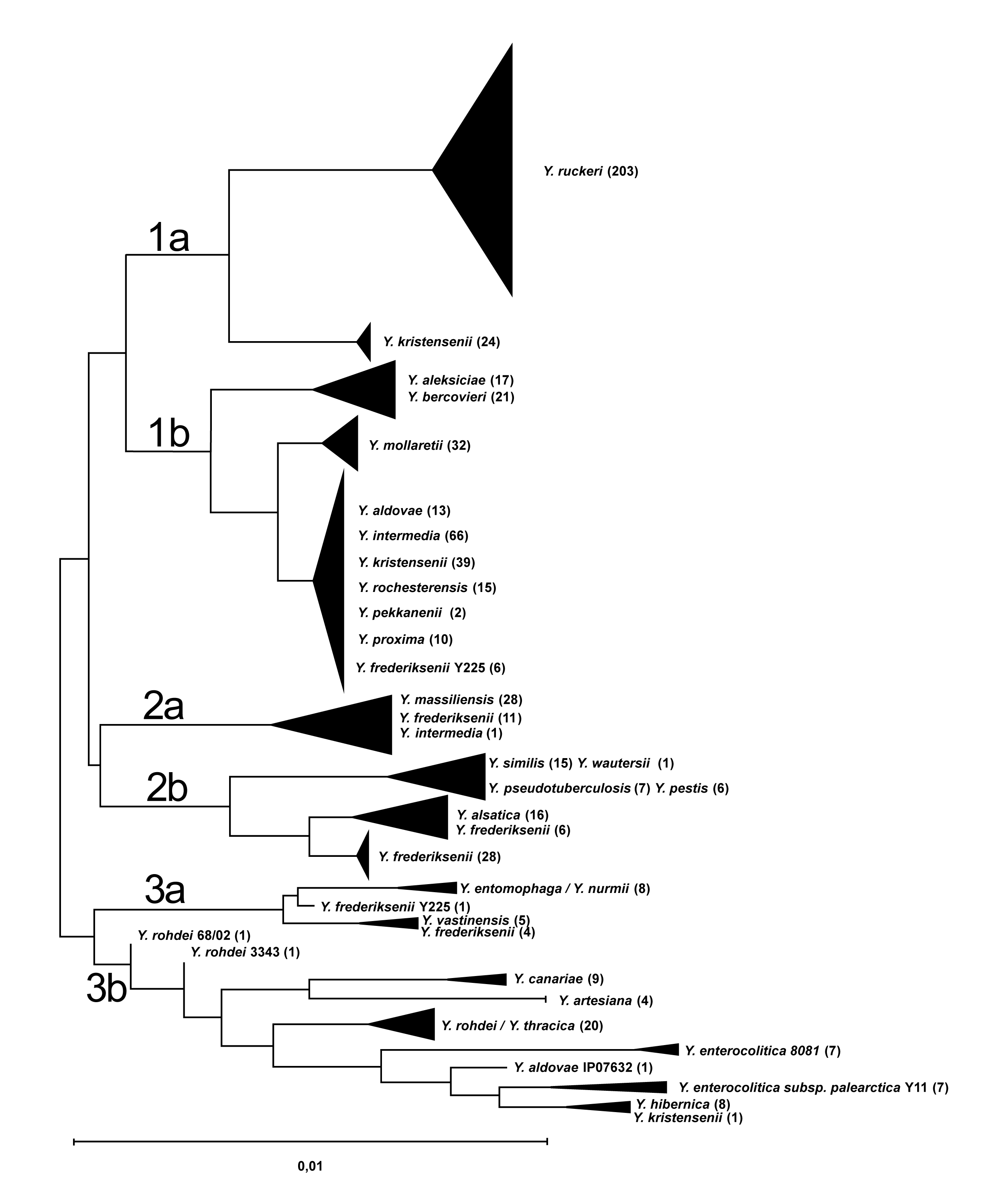

Supplement: SUPPLEMENTARY FIGURE S1 — Neighbor-joining phylogenetic tree based on 644 aligned genes of the 16S rRNA genus Yersinia. [file Image_1.tif]
