# Supplementary material for: Evaluation of 16S rRNA genes sequences and genome-based analysis for identification of non-pathogenic Yersinia
Source: Front Microbiol. 2025 Jan 7;15:1519733. doi: 10.3389/fmicb.2024.1519733 (PMC11753223; doi:10.3389/fmicb.2024.1519733)

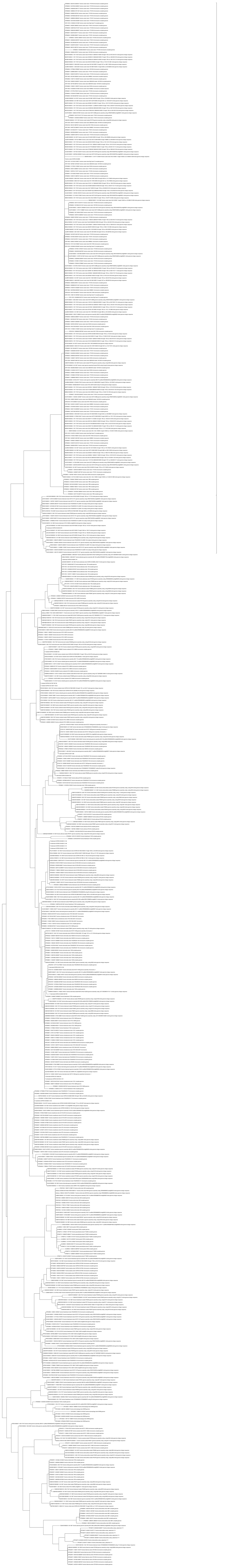

Supplement: SUPPLEMENTARY FIGURE S3 — Neighbor joining tree based on 9494 core SNPs in Yersinia genomes. This tree was rooted using midpoint option. The groups of genomes observed in the tree were identified according to the species names of the type strains. [file Data_Sheet_1.pdf]
